# Biopsy-free in vivo virtual histology of skin using deep learning

**DOI:** 10.1038/s41377-021-00674-8

**Published:** 2021-11-18

**Authors:** Jingxi Li, Jason Garfinkel, Xiaoran Zhang, Di Wu, Yijie Zhang, Kevin de Haan, Hongda Wang, Tairan Liu, Bijie Bai, Yair Rivenson, Gennady Rubinstein, Philip O. Scumpia, Aydogan Ozcan

**Affiliations:** 1 Electrical and Computer Engineering Department, University of California, Los Angeles, CA 90095 USA; 2 Bioengineering Department, University of California, Los Angeles, CA 90095 USA; 3 California NanoSystems Institute (CNSI), University of California, Los Angeles, CA 90095 USA; 4 Dermatology and Laser Centre, Studio City, CA 91604 USA; 5 Computer Science Department, University of California, Los Angeles, CA 90095 USA; 6 Division of Dermatology, University of California, Los Angeles, CA 90095 USA; 7 Department of Dermatology, Veterans Affairs Greater Los Angeles Healthcare System, Los Angeles, CA 90073 USA; 8 Department of Surgery, University of California, Los Angeles, CA 90095 USA

**Keywords:** Confocal microscopy, Biophotonics, Imaging and sensing

## Abstract

An invasive biopsy followed by histological staining is the benchmark for pathological diagnosis of skin tumors. The process is cumbersome and time-consuming, often leading to unnecessary biopsies and scars. Emerging noninvasive optical technologies such as reflectance confocal microscopy (RCM) can provide label-free, cellular-level resolution, in vivo images of skin without performing a biopsy. Although RCM is a useful diagnostic tool, it requires specialized training because the acquired images are grayscale, lack nuclear features, and are difficult to correlate with tissue pathology. Here, we present a deep learning-based framework that uses a convolutional neural network to rapidly transform in vivo RCM images of unstained skin into virtually-stained hematoxylin and eosin-like images with microscopic resolution, enabling visualization of the epidermis, dermal-epidermal junction, and superficial dermis layers. The network was trained under an adversarial learning scheme, which takes ex vivo RCM images of excised unstained/label-free tissue as inputs and uses the microscopic images of the same tissue labeled with acetic acid nuclear contrast staining as the ground truth. We show that this trained neural network can be used to rapidly perform virtual histology of in vivo, label-free RCM images of normal skin structure, basal cell carcinoma, and melanocytic nevi with pigmented melanocytes, demonstrating similar histological features to traditional histology from the same excised tissue. This application of deep learning-based virtual staining to noninvasive imaging technologies may permit more rapid diagnoses of malignant skin neoplasms and reduce invasive skin biopsies.

## Introduction

Microscopic evaluation of histologically processed and chemically stained tissue is the gold standard for the diagnosis of a wide variety of medical diseases. Advances in medical imaging techniques, including magnetic resonance imaging, computed tomography, and ultrasound, have transformed medical practice over the past several decades, decreasing the need for invasive biopsies and exploratory surgeries. Similar advances in imaging technologies to aid in the diagnosis of skin disease noninvasively have been slower to progress.

Skin cancers represent the most common type of cancer diagnosed in the world. Basal cell carcinoma (BCC) comprises 80% of the 5.4 million skin cancers seen in the United States annually^1^. Melanoma represents a small percentage of overall skin cancers but represents the leading cause of death from skin cancer and is among the deadliest cancers when identified at advanced stages^2^. Invasive biopsies to differentiate BCC from benign skin neoplasms and melanoma from benign melanocytic nevi represent a large percentage of the biopsies performed globally. Over 8.2 million skin biopsies are performed to diagnose over 2 million skin cancers annually in the Medicare population alone^1^, resulting in countless unnecessary biopsies and scars at a high financial burden. In addition, the process of biopsy, histological tissue processing, delivery to pathologists, and diagnostic assessment requires one day to several weeks for a patient to receive a final diagnosis, resulting in lag time between the initial assessment and definitive treatment. Thus, noninvasive imaging presents an opportunity to prevent unnecessary skin biopsies while improving the early detection of skin cancer^3^.

The most used ancillary optical imaging tool used by dermatologists are dermatoscopes, which magnify skin lesions and use polarized light to assess superficial features of skin disease and triage lesions with ambiguous features for tissue biopsy^4^. While dermatoscopes can reduce biopsies in dermatology, their use requires proper training to improve the sensitivity of detecting skin cancers over clinical inspection alone^5^. More advanced optical technologies have been developed for noninvasive imaging of skin cancers, including reflectance confocal microscopy (RCM), optical coherence tomography (OCT), multiphoton microscopy (MPM), and Raman spectroscopy, among others^6,7^. Of these optical imaging technologies, only RCM and MPM technologies provide cellular-level resolution similar to tissue histology and allow for better correlation of image outputs to histology due to their ability to discern cellular-level details.

RCM imaging detects backscattered photons that produce a grayscale image of tissue based on the contrast of relative variations in refractive indices and sizes of organelles and microstructures^8,9^. Currently, RCM can be considered as the most clinically-validated optical imaging technology with strong evidence supporting its use by dermatologists to discriminate benign from malignant lesions with high sensitivity and specificity^10,11^. Importantly, several obstacles remain for accurate interpretation of RCM images, which requires extensive training for novice readers^12^. While the black and white contrast images can be used to distinguish types of cells and microstructural detail, in vivo RCM does not show nuclear features of skin cells in a similar fashion to the traditional microscopic evaluation of tissue histology. Multimodal ex vivo fluorescence and RCM can produce digitally-colorized images with nuclear morphology using fluorescent contrast agents^13,14^. However, these agents are not used in vivo with a reflectance-based confocal microscopy system. Without nuclear contrast agents, nuclear features critical for assessing cytologic atypia are not discernable. Further, the grayscale image outputs and horizontal imaging axis of confocal technologies pose additional challenges for diagnosticians who are accustomed to interpreting tissue pathology with nuclear morphology in the vertical plane. Combined, these visualization-based limitations, in comparison to standard-of-care biopsy and histopathology, pose barriers to the wide adoption of RCM.

On the other hand, hematoxylin and eosin (H&E) staining of tissue sections on microscopy slides represents the most common visualization format used by dermatologists and pathologists to evaluate skin pathology. Thus, conversion of images obtained by noninvasive skin imaging and diagnostic devices to an H&E-like format may improve the ability to diagnose pathological skin conditions by providing a virtual “optical biopsy” with cellular resolution and in an easy-to-interpret visualization format.

Deep learning represents a promising approach for computationally-assisted diagnosis using images of skin. Deep neural networks trained to classify skin photographs and/or dermoscopy images, successfully discriminated benign from malignant neoplasms at a similar accuracy to trained dermatologists^15,16^. Algorithms based on deep neural networks can help pathologists identify important regions of disease, including microscopic tumor nodules, neoplasms, fibrosis, inflammation, and even allow prediction of molecular pathways and mutations based on histopathological features^17–22^. Researchers also used deep neural networks to perform semantic segmentation of different textual patterns in RCM mosaic images of melanocytic skin lesions as a potential diagnostic aid for clinicians^23,24^. Apart from these histopathology-based dermatology applications, deep learning has also been used in other biomedical microscopic imaging applications, such as super-resolution^25^, digital refocusing^26^, nuclei segmentation^27^, quantitative phase imaging with computational interference microscopy^28^, and label-free virtual histopathology enabled by multiphoton microscopy^29^, among others. Deep learning-based approaches have also enabled the development of algorithms to learn image transformations between different microscopy modalities to digitally enhance pathological interpretation. For instance, using unstained, autofluorescence images of label-free tissue sections, a deep neural network can virtually stain images of the slides, digitally matching the brightfield microscopy images of the same samples stained with standard histochemical stains such as H&E, Jones, Masson’s Trichrome, and periodic acid Schiff (PAS) without the need for histochemical processing of tissue^30–32^. These virtually-stained images were found to be statistically indiscernible to pathologists when compared in a blinded fashion to the images of the chemically stained slides^30^. Deep learning-enabled virtual staining of unstained tissue has been successfully applied to other types of label-free microscopic imaging modalities including e.g., quantitative phase imaging^33^ and two-photon excitation with fluorescence lifetime imaging^34^, but has not been used to obtain in vivo virtual histology.

Here, we describe a novel, deep learning-based tissue staining framework to rapidly perform in vivo virtual histology of unstained skin. In the training phase of this framework, we used RCM images of excised skin tissue with and without acetic acid nuclear contrast staining to train a deep convolutional neural network (CNN) using structurally-conditioned generative adversarial networks (GAN)^35,36^, together with attention gate modules that process three-dimensional (3D) spatial structure of tissue using 3D convolutions. First, we acquired time-lapse RCM image stacks of ex vivo skin tissue specimens during the acetic acid staining process to label cell nuclei. Using this 3D data, label-free, unstained image stacks were accurately registered to the corresponding acetic acid-stained 3D image stacks, which provided a high degree of spatial supervision for the neural network to map 3D features in label-free RCM images to their histological counterparts. Once trained, this virtual staining framework was able to rapidly transform in vivo RCM images into virtually stained, 3D microscopic images of normal skin, BCC, and pigmented melanocytic nevi with H&E-like color contrast. When compared to traditional histochemically-processed and stained tissue sections, our digital technique demonstrates similar morphological features that are observed in H&E histology. In vivo virtual staining of unprocessed skin through noninvasive imaging technologies such as RCM would be transformative for rapid and accurate diagnosis of malignant skin neoplasms, also reducing unnecessary skin biopsies.

## Results

### Training of virtual staining networks for in vivo histology of unstained skin

**Fig. 1:**
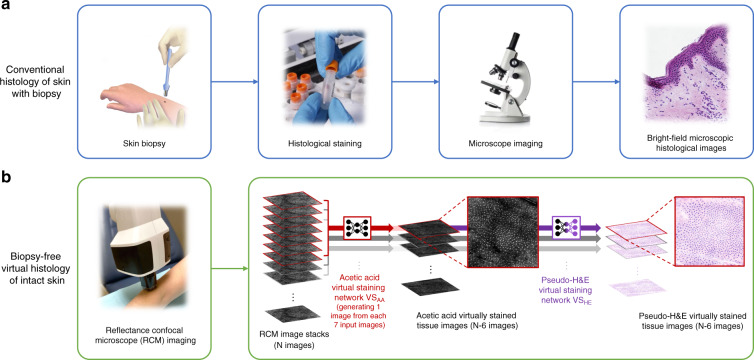
The schematic diagram demonstrating the conventional (top) and biopsy-free virtual (bottom) histological staining procedures for skin pathology. (**a**) Standard tissue biopsy, followed by tissue fixation, processing, and staining results in microscopy slides for pathological interpretation. (**b**) By employing the trained deep neural network that takes a stack of RCM images of unstained intact skin as input and instantly generates corresponding virtually stained tissue images, the reported deep learning-based virtual histology of skin may provide a unique avenue to biopsy-free, label-free clinical dermatological diagnosis. Each time, a stack of seven axially adjacent RCM images is fed into a trained deep neural network VS_AA_ and transformed into an acetic acid virtually stained tissue image that is corresponding to the central image of the input stack, so that a stack of *N* images can be used to generate *N*-6 virtually stained 3D output images that are axially adjacent. Following this acetic acid virtual staining, a pseudo-H&E virtual staining step is further performed by a trained deep neural network (VS_HE_).

A traditional biopsy requires cleansing and local anesthesia of the skin, followed by surgical removal, histological processing, and examination by a trained physician in histopathological assessment, typically using H&E staining, as depicted in Fig. 1a. Through the combination of two subcomponents, i.e., hematoxylin and eosin, this staining method is able to stain cell nuclei blue and the extracellular matrix and cytoplasm pink, so that clear nuclear contrast can be achieved to reveal the distribution of cells, providing the foundation for the evaluation of the general layout of the skin tissue structure. In our Results, we demonstrate a new approach using deep learning-enabled transformation of label-free RCM images into H&E-like output images, without the removal of tissue or a biopsy, as illustrated in Fig. 1b. Current standard formats of RCM imaging of skin include obtaining stacks of images through different layers of the skin and obtaining a mosaic image through one of the layers of skin. We believe that the combination of these two formats could provide abundant information input for 3D skin virtual histology. However, obtaining H&E images of the same skin tissue to establish the ground truth for network training is a major challenge. Directly using the brightfield microscopy images of the histochemically-stained (H&E) tissue slides after the biopsy as our ground truth is simply not feasible, because H&E staining requires a series of operations, including biopsy, sectioning, and chemical processing, all of which bring severe deformations to the tissue structure and create major difficulties in aligning the H&E-stained tissue images with the in vivo RCM images of the unstained skin. Furthermore, direct in vivo RCM imaging of unstained skin is unable to provide the demanded nuclear contrast at the input of the network.

**Fig. 2:**
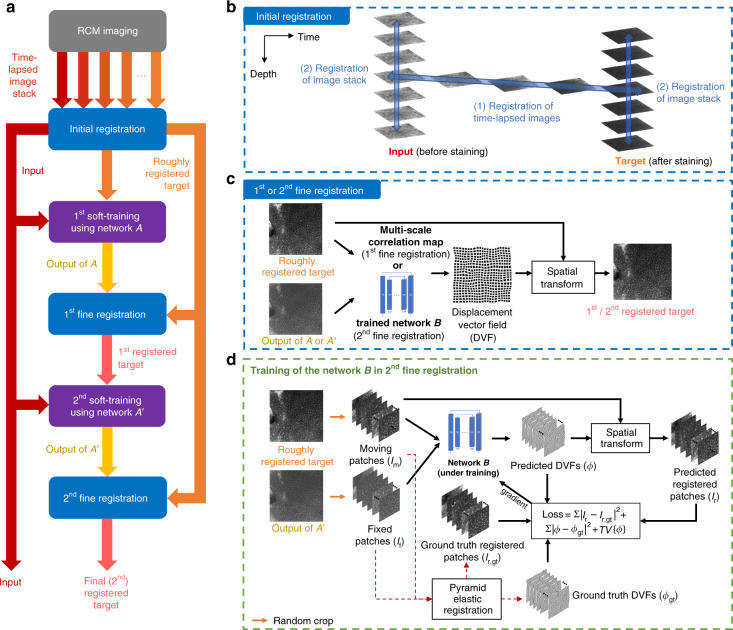
Image registration process for generating input-target image pairs for the training phase. (**a**–**d**) illustrate the details of the image registration workflow. See the “Image preprocessing and registration” section in Materials and Methods for further details.

**Fig. 3:**
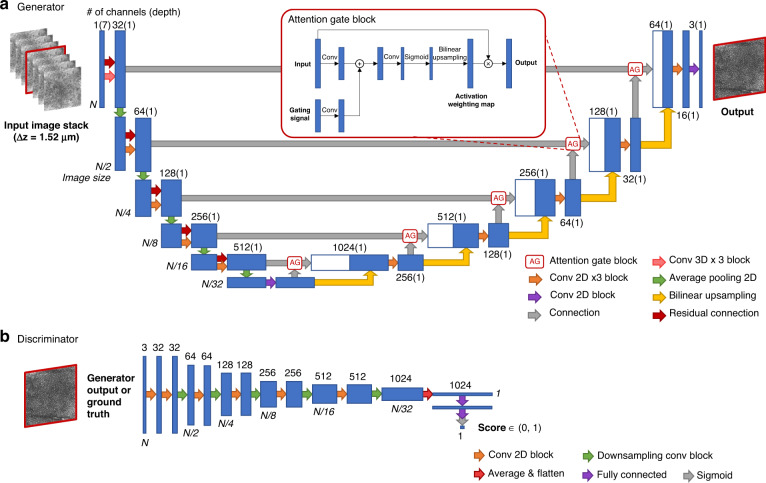
Architecture of the GAN-based deep neural network. (**a**) Generator. (**b**) Discriminator. See the “Network architecture and training schedule” section in Materials and Methods for details.

Inspired by the fact that acetic acid was used to provide nuclear contrast in RCM imaging of cervical tissue^37^ and Mohs surgical skin excisions^38,39^, we reasoned that the same reagent can also be used to quickly stain the ex vivo skin tissue in RCM imaging, bringing nuclear contrast to serve as our ground truth. We performed the training experiments accordingly and took time-lapsed RCM videos in the process of acetic acid staining, through which we obtained the 3D image sequences with feature positions traceable before and after the acetic acid staining. According to these sequences, we initially performed a rough registration of the images before and after staining, which was followed by two more rounds of deep learning-based fine image registration processes to obtain accurately registered image stacks, as shown in Fig. 2. These registered image stacks were then used for the training of the acetic acid virtual staining network named VS_AA_, where attention gate modules and 3D convolutions are employed to enable the network to better process the 3D spatial structure of tissue; see Fig. 3. For generating the in vivo image stack with acetic acid virtual staining, for each inference, VS_AA_ takes a stack of seven axially-adjacent RCM images of horizontal cross-sections of unstained skin tissue and outputs the virtually stained tissue image that is corresponding to the central image of the input stack, which forms a “7-to-1” image transformation; see Fig. 1b. Based on this scheme, by processing all the *N* input RCM images in the input stack, the network VS_AA_ generates a virtually stained 3D image stack that is composed of *N*-6 output images. We trained VS_AA_ using the aforementioned registered image stacks with a training set composed of 1185 input/output image pairs and also transformed the acetic acid virtual staining results into H&E-like images using another, trained deep neural network, named pseudo-H&E virtual staining network: VS_HE_, as illustrated in Fig. 1b. More details about the image registration process, network structure, and the training details of acetic acid and pseudo-H&E virtual staining networks (i.e., VS_AA_ and VS_HE_, respectively) can be found in the Materials and Methods section.

### Virtual staining of RCM image stacks of normal skin samples ex vivo

**Fig. 4:**
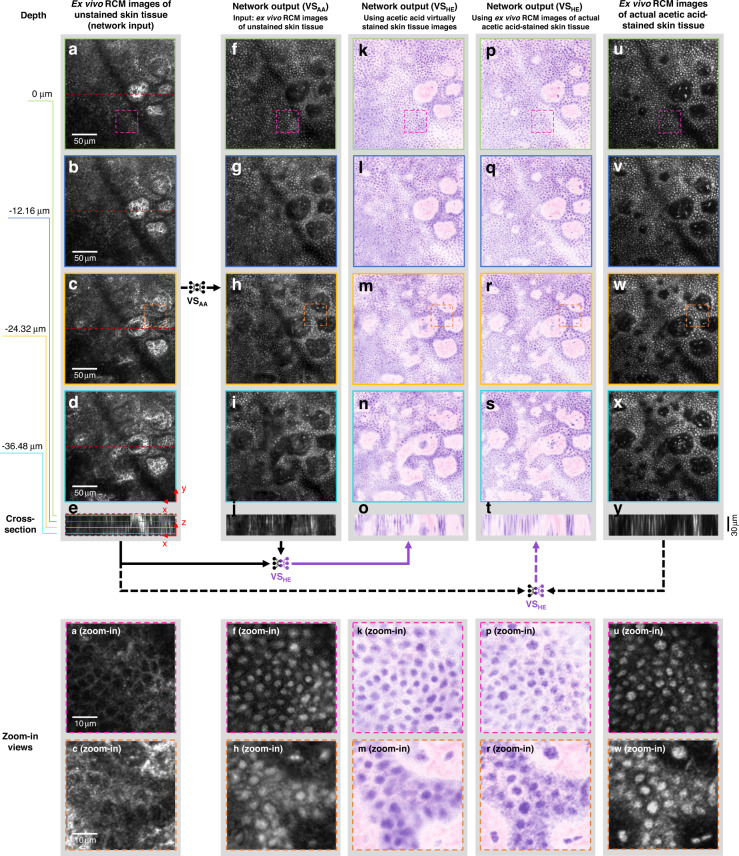
3D ex vivo virtual staining results of a skin tissue area around the dermal-epidermal junction and their comparison with ground truth, actual acetic acid staining. **a**–**d** Label-free RCM images showing an ex vivo skin tissue area at different depths around dermal-epidermal junction without any staining, served as the network inputs. The depth of (**b**), (**c**), and (**d**) were 12.16, 24.32, and 36.48 μm below **a** into the skin, respectively. **e** Cross-section of the RCM image stack of the tissue area including (**a**–**d**). Lines in different colors are used to indicate the depth positions of (**a**–**d**). **f**–**i** Acetic acid virtual staining results of the same tissue area and depth as (**a**–**d**) generated by the deep neural network VS_AA_. **j** is the image stack cross-section of the acetic acid virtual staining results including (**f**–**i**) generated using the acetic acid virtually stained tissue images. **k**–**n** Pseudo-H&E virtual staining results generated using the acetic acid virtually stained tissue images (**f**–**i**). These H&E-like images were generated by the pseudo-H&E virtual staining network VS_HE_ that took both the RCM images of the unstained tissue (**a**–**d**) and acetic acid virtually stained tissue images (**f**–**i**) as input (see solid arrows below the upper panel). **o** Cross-section of the pseudo-H&E virtually stained tissue image stack including (**k**–**n**). **u**–**x** RCM images of the same tissue area and depth as (**a**–**d**) after the actual acetic acid staining process, served as ground truth for (**f**–**i**). **y** shows the cross-section of the image stack of the tissue stained with acetic acid including (**u**–**x**). **p**–**s** Pseudo-H&E virtual staining results generated using the actual acetic acid-stained images (**u**–**x**). These H&E-like images were generated by the same pseudo-H&E virtual staining network VS_HE_ that took the RCM images of the unstained tissue (**a**–**c**) and actual acetic acid-stained images (**q**–**s**) as input (see dashed arrows below the upper panel and see Materials and Methods for more details). **t** shows the cross-section of the pseudo-H&E virtually stained tissue image stack including (**p**–**s**) generated using the actual acetic acid-stained images. Zoomed-in views of some portions of the images are provided at the bottom for a better visual comparison of details.

Staining of skin blocks with acetic acid allowed the visualization of nuclei from excised tissue at the dermal-epidermal junction and superficial dermis in normal skin samples. Using these images as our ground truth (only for comparison), we first tested whether the RCM images of unstained tissue can be transformed into H&E-like images using the deep learning-based virtual histology method. Our data, summarized in Fig. 4, demonstrate that cross-sections of RCM image stacks taken at various depths around the dermal-epidermal junction of a skin lesion could be transformed into virtually stained tissue images with inferred nuclei, showing good correspondence with the actual acetic acid-stained RCM images used for ground truth comparison. Furthermore, we performed pseudo-H&E virtual staining using these acetic acid-stained image results, as shown in Fig. 4. An example of traditionally processed skin histology through the dermal-epidermal junction in the horizontal plane is also shown in Fig. S1a to illustrate the visual similarity of the virtually stained tissue image shown in Fig. 4. The acetic acid virtual staining network VS_AA_ performed similarly well when ex vivo image stacks of the spinous layer of the epidermis were utilized as input, as shown in Fig. S2.

**Fig. 5:**
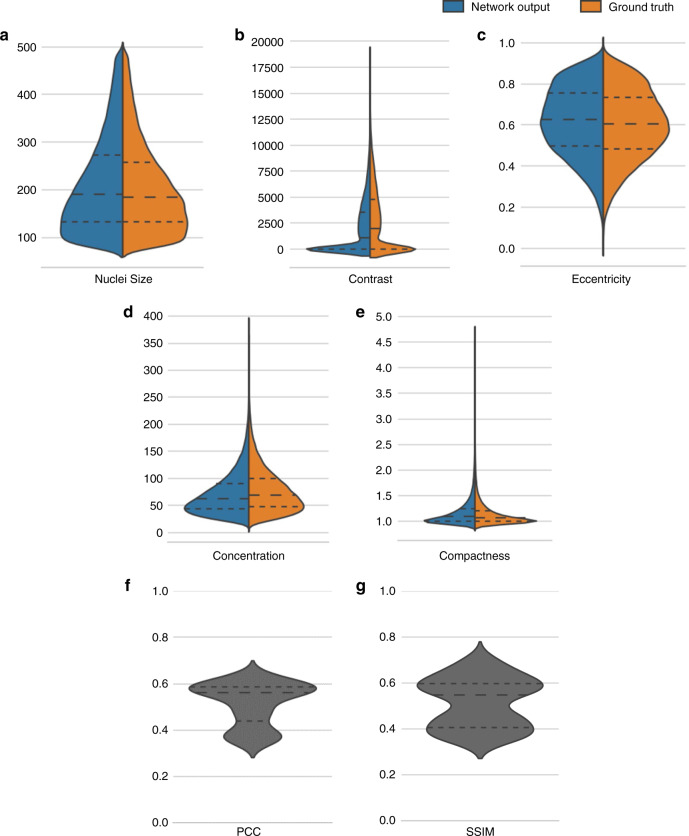
Quantitative analysis of the acetic acid virtual staining results on ex vivo skin tissue samples. **a–e** Violin plots show quantitative comparisons of the statistical distribution of the measured nuclear morphological parameters between the acetic acid virtually stained skin tissue images (blue) and their corresponding ground truth images obtained using actual acetic acid staining (orange). Five metrics are used for the comparison: **a** nuclear size, **b** contrast, **c** eccentricity, **d** concentration, and **f** compactness (see Materials and Methods for details). The statistical results cover a total number of 96,731 nuclei, detected in 176 ex vivo tissue images of normal skin. **f**, **g** Violin plot shows the statistical distribution of the PCC and SSIM values measured through comparing the virtually stained (acetic acid) tissue images against their corresponding actual acetic acid-stained ground truth images. In all the violin plots presented above, the dashed lines from top to bottom represent the 75, 50 (median), and 25 quartiles, respectively.

Next, we evaluated the prediction performance of our model through a series of quantitative analyses. To do so, first, we generated the acetic acid virtual staining results of the entire ex vivo testing set that contains 199 ex vivo RCM images collected from six different unstained skin samples from six patients. We performed segmentation on both the virtual histology images of normal skin samples and their ground truth images to identify the individual nuclei on these images. Using the overlap between the segmented nuclear features of acetic acid virtual staining images and those in the actual acetic acid-stained ground truth images as a criterion, we classified each nucleus in these images into the categories of true positive (TP), false positive (FP), and false negative (FN) and quantified the sensitivity and precision values of our prediction results (see Materials and Methods for details). We found that our virtual staining results achieved ~80% sensitivity and ~70% precision for nuclei prediction on the ex vivo testing image set. Then, using the same segmentation results, we further assessed the nuclear morphological features in the acetic acid virtual staining and ground truth images. Five morphological metrics, including nuclear size, eccentricity, compactness, contrast, and concentration, were measured for this analysis (see Materials and Methods for details). As shown in Fig. 5a–e, these analyses demonstrate that the statistical distributions of these nuclear morphological parameters calculated using the acetic acid virtual staining results presented a very good match with those of the actual acetic acid-stained ground truth images, regardless of the metrics used. In order to further demonstrate the efficacy of our virtual staining results for three-dimensional imaging, in Fig. S3 we also report the results of the same type of analysis for the image stack used and shown in Fig. 4, but this time focusing on different depth ranges within the tissue block: once again, a strong match between the acetic acid virtually stained skin tissue images and their actual acetic acid-stained ground truth is observed for all the quantitative metrics used, regardless of the depth range selected. In addition, to evaluate our results from the perspective of overall image similarity, we also calculated the Pearson correlation coefficient (PCC) and the structural similarity index (SSIM)^40^ of each image pair composed of acetic acid virtual staining results and the ground truth in the ex vivo testing image set. The results of these PCC and SSIM analyses are reported in Fig. 5f–g, where the median PCC and SSIM values are found to be 0.561 and 0.548, respectively.

### Virtual staining of RCM image stacks of melanocytic nevi and basal cell carcinoma ex vivo

**Fig. 6:**
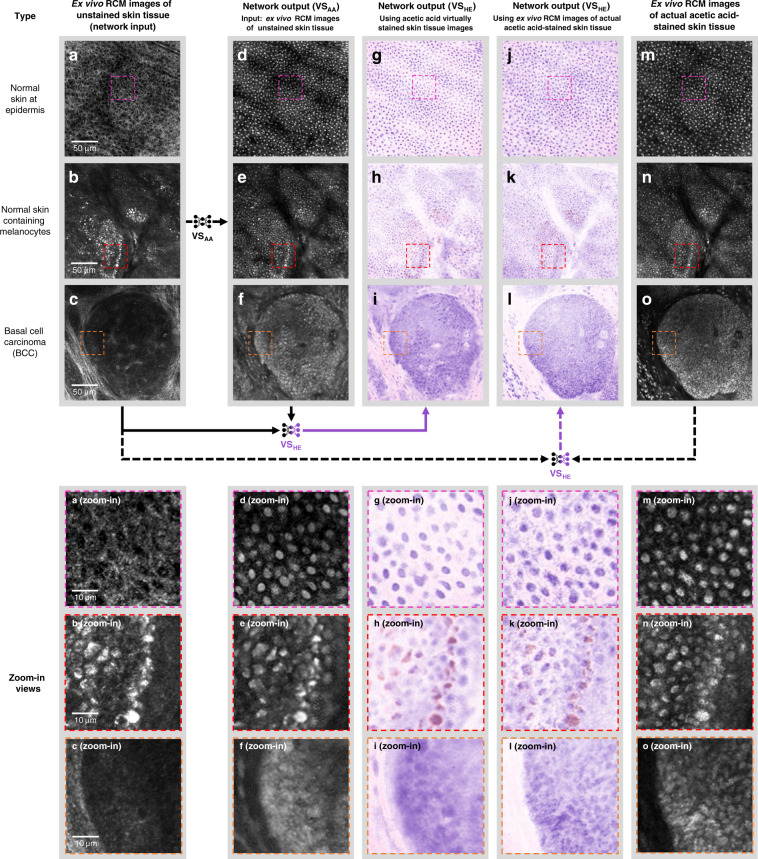
Virtual staining results for different types of ex vivo skin tissue areas and their comparison with ground truth, actual acetic acid staining. **a–c** Label-free RCM images of three different types of ex vivo skin tissue areas, including **a** normal skin, **b** a melanocytic nevus, and **c** skin containing BCC, which are used as input of the virtual staining neural networks. **d–f** Acetic acid virtual staining results of the same tissue areas in (**a**–**c**) generated by the deep neural network VS_AA_. **g–i** Pseudo-H&E virtual staining results generated using the acetic acid virtually stained tissue images (**d**–**f**). These H&E-like images were generated by the pseudo-H&E virtual staining network VS_HE_ that took both the RCM images of the unstained tissue (**a**–**c**) and the acetic acid virtually stained tissue images (**e**–**g**) as input (see solid arrows below the upper panel). **m**–**o** RCM images of the same tissue area and depth as (**a**–**c**) after the actual acetic acid staining process, which served as ground truth for (**d**–**f**). **j**–**l** Pseudo-H&E virtual staining results generated using the actual acetic acid-stained images (**m**–**o**). These H&E-like images were generated by the same pseudo-H&E virtual staining network VS_HE_ that took the RCM images of the unstained tissue (**a**–**c**) and the actual acetic acid-stained images (**m**–**o**) as input (see the dashed arrows below the upper panel and the Materials and Methods section for details). Zoomed-in views of some portions of the images are provided at the bottom for a better visual comparison of details.

To determine whether the presented method can be used to assess skin pathology, we imaged features seen in common skin neoplasms. Melanocytes are found at the dermal-epidermal junction in normal skin and increase in number and location in both benign and malignant melanocytic neoplasms. For our approach to be successful, it needs to incorporate pigmented melanocytes in order to be useful for the interpretation of benign and malignant melanocytic neoplasms (nevi and melanoma, respectively). Melanin provides strong endogenous contrast in melanocytes during RCM imaging without acetic acid staining^8^. This allows melanocytes to appear as bright cells in standard RCM images due to the high refractive index of melanin^8^. We compared specimens with normal proportions of melanocytes, shown in Fig. 6, the first row, to specimens containing abundant melanocytes, such as benign melanocytic nevi shown in Fig. 6, second row. Our pseudo-H&E virtual staining algorithm was able to successfully stain melanocytes and provide pigment coloration similar to the brown pigment seen on histologically-stained specimens. An example of a histologically-stained skin tissue section image with brown pigment is provided in Fig. S1b.

Unlike melanocytes, basaloid cells that comprise tumor islands in BCC appear as dark areas in RCM images^41^. This appearance is due to the high nuclear to cytoplasmic ratio seen in malignant cells and the fact that nuclei do not demonstrate contrast on RCM imaging. Further, mucin present within and surrounding basaloid islands in BCC further limits the visualization of tumor islands due to a low reflectance signal. Since many skin biopsies are performed to rule out BCC, we next determined whether acetic acid staining can provide ground truth for skin samples containing BCC. 50% acetic acid concentration allowed sufficient penetration through the mucin layer to stain nuclei of BCC. We used discarded, ~2 mm thick, Mohs surgical specimens diagnosed as BCC and performed RCM imaging without and with acetic acid staining (the latter formed the ground truth). As illustrated in the third row of Fig. 6, our virtual staining results showed strong concordance of features of BCC when compared to these acetic acid-stained ground truth images; common histological features of BCC, including islands of basaloid cells with small, peripherally palisaded nuclei and dark silhouettes^42,43^, a material resembling mucin within the basaloid islands, and separation (retraction) of basaloid islands from the surrounding stroma were visible in the virtually stained RCM images containing BCC as shown in Fig. 6, third row.

### Virtual staining of mosaic RCM images ex vivo

Mosaic images are formed by multiple individual RCM images scanned over a large area at the same depth to provide a larger field of view of the tissue to be examined for interpretation and diagnosis. To demonstrate virtual staining of mosaic RCM images, ex vivo RCM images of BCC in a tissue specimen obtained from a Mohs surgery procedure were converted to virtual histology. Through visual inspection, the virtual histology image shown in Fig. S4 demonstrated similar features observed in a representative histological section (not in the same plane as the RCM images) obtained from the actual frozen section histology of the processed tissue. Of note, this specimen used for Fig. S4 displayed both nodular and infiltrative islands of BCC. Since our algorithm was primarily trained on nodular and superficial types of BCC, it is not surprising that it performed much better at revealing the nodular islands of BCC (marked with yellow asterisks in Fig. S4c, d) within the specimen, rather than the thin anastomosing cords of infiltrative BCC displaying keratinization (pink/eosinophilic appearance in the light blue dotted regions in Fig. S4c, d), although both nodules and individual thin cords are still visible in the virtually stained image shown in Figure S4c.

### Virtual staining of in vivo image stacks and mosaic RCM images

**Fig. 7:**
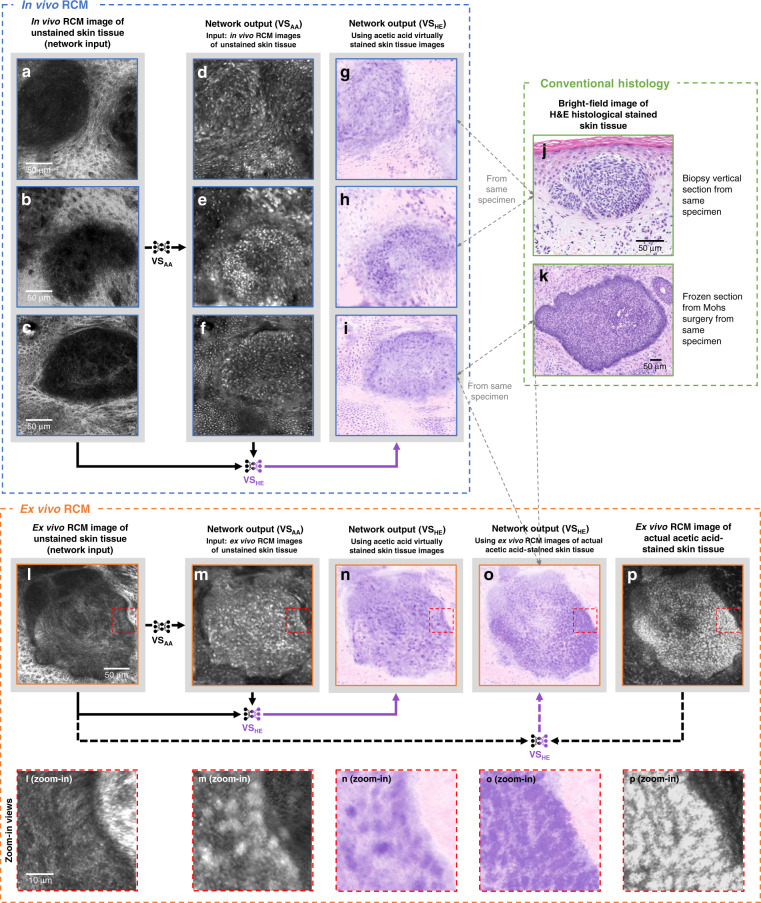
Virtual staining results of in vivo RCM images of skin tissue areas that contain BCC. **a**–**i** are in vivo RCM images of unstained skin, while **j**, **k** and **l**–**p** are H&E histology and ex vivo RCM images used for comparison, respectively. Our trained network VS_AA_ transformed label-free in vivo RCM images of unstained tissue areas with BCC (**a**–**c**) as input into their acetic acid virtual staining results (**d**–**f**). Pseudo-H&E virtual staining was further performed by the trained network VS_HE_ to generate the H&E versions of (**d**–**f**) by taking both the RCM images of the unstained tissue (**a**–**c**) and the acetic acid virtually stained tissue images (**d**–**f**) as input (see arrows at the bottom of the blue panel). For comparison with these in vivo virtual staining results, in (**j**) and (**k**) we show bright-field images of visually similar BCC regions taken from the same specimen after H&E histochemical staining. Note that these BCC regions (**g**–**i**) are not necessarily the same BCC tumor nodule as shown in H&E histology (**j**–**k**), but are from the same specimen, and may be subject to structural deformations due to the standard histochemical staining and related sample processing. As the gray dashed arrows indicate, **j** is the H&E histology of a vertical section biopsy taken from the same specimen used for (**g**, **h**), and **k** is the H&E histology of a frozen section from Mohs surgery taken from the same specimen used for in vivo (**i**) and ex vivo (**o**). As another comparison, we also show ex vivo acetic acid virtually stained and actual acetic acid-stained results for the same specimen used for (**i**). We used the same trained network VS_AA_ to transform label-free ex vivo RCM images of unstained tissue areas with BCC (**l**) into ex vivo acetic acid virtually stained tissue images shown in (**m**), forming a comparison with the ground truth images of the same tissue area actually stained with acetic acid (**p**). The same pseudo-H&E virtual staining was also applied to (**m**, **p**) using the network VS_HE_ to generate their pseudo-H&E virtually stained counterparts (**n**, **o**) (see the arrows at the bottom of the orange panel and see Materials and Methods for details). Zoomed-in views of some portions of the ex vivo RCM images are provided at the bottom of the orange panel for a better visual comparison of details.

Next, we tested whether RCM images of unstained skin obtained in vivo can give accurate histological information using our trained neural network. We compared in vivo RCM images of lesions that are suspicious for BCC to (1) histology from the same lesion obtained following biopsy or Mohs section histology and (2) images obtained ex vivo with acetic acid staining (ground truth). As summarized in Fig. 7, virtual staining of in vivo RCM images shown in Fig. 7g–i again demonstrated features compatible with BCC tumor islands commonly seen on histologically processed and stained tissue; see Fig. 7j, k. These results were further confirmed with the ex vivo RCM image of the actual acetic acid-stained tissue of the same lesion, as shown in Fig. 7o, p. The virtual histology output from the trained algorithm using the in vivo images of the skin lesion displayed similar basaloid tumor islands as those seen in the actual acetic acid-stained ex vivo RCM images and the actual histology. We also present other examples of in vivo stacks of RCM images of normal skin, a junctional nevus, and another BCC sample in Fig. S5. The junctional nevus showed expansion of melanocytic cells at the dermal-epidermal junction in a benign ringed pattern. One plane of the image stack is shown for these samples. Another sample, reported in Fig. S6, shows various planes of a confocal stack of a junctional nevus through all of the skin layers including the granular layer (first row), spinous layer (second row), basal layer (third row), and dermal-epidermal junction (fourth row).

**Fig. 8:**
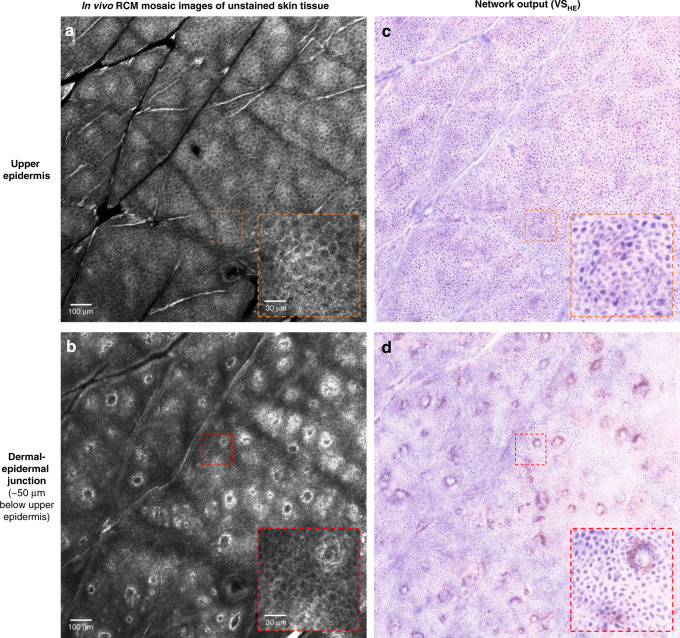
Pseudo-H&E virtual staining results of large field-of-view mosaic images of an in vivo skin tissue at two different depths. **a**, **b** Label-free in vivo RCM image mosaic at two cross-sections corresponding to **a** upper epidermis and **b** dermal-epidermal junction. The axial gap between the two cross-sections is around 50 μm. **c**, **d**, Pseudo-H&E virtual staining results of (**a**) and (**b**), respectively

We also examined whether our virtual staining method can be applied to mosaic in vivo RCM images, despite the fact that the network was not trained on a full set of mosaic images. These mosaic RCM images are important because they are often used in clinical settings to extend the field of view for interpretation and are required for the reimbursement of the RCM imaging procedure. Our results reported in Fig. 8 reveal that in vivo mosaic images of unstained skin tissue, through the spinous layer of the epidermis and the dermal-epidermal junction, were successfully transformed into H&E-like images without acetic acid staining. These results confirm that the virtual staining network trained on confocal image stacks was able to perform virtual in vivo histology of RCM image stacks of common skin lesions, including BCC and nevus, as well as large mosaic RCM images of normal skin without the need for further training.

Finally, we tested the inference speed of our trained deep network models using RCM image stacks and demonstrated the feasibility of real-time virtual staining operation (see Materials and Methods for details). For example, using eight Tesla A100 GPUs to perform virtual staining through VS_AA_ and VS_HE_ networks, the inference time for an image size of 896 × 896-pixels was reduced to ~0.0173 and ~0.0046 s, respectively. Considering the fact that the frame rate of the RCM device we used is ~9 frames per second (~0.111 sec/image), this demonstrated virtual staining speed is sufficient for real-time operation in clinical settings.

## Discussion

Previous studies have used machine learning and deep neural networks to differentiate benign from malignant lesions of the skin from e.g., clinical photographs, dermatoscopic images, and multispectral imaging, to provide a computer-assisted diagnosis. In this study, we applied a deep neural network-based approach to perform virtual staining in RCM images of label-free normal skin, BCC, and melanocytic nevi. We also transformed grayscale RCM images into pseudo-H&E virtually stained images that resembled H&E staining, the visualization format most commonly used by pathologists to assess biopsies of histochemically-stained tissue on microscopy slides.

In our virtual staining inference, we used a 3D image stack as the input of the GAN model. We conducted an ablation study to demonstrate that using 3D RCM image stacks, composed of seven adjacent images, is indeed necessary for preserving the quality of the acetic acid virtual staining results. For this comparative analysis, we changed the input of our network VS_AA_ to only one RCM image of unstained skin tissue that was located at the same depth as the actual acetic acid-stained target (ground truth image). Then, we trained a new VS_AA_ without having a major change to its structure, except that the first three 3D convolutions were changed to 2D (see Fig. 3 for the original network structure). Compared to acetic acid virtual staining results that we have got using 3D RCM image stacks as input, the results that used a single 2D RCM image as input produced suboptimal results that were significantly blurred (see Fig. S7). The reason for this degradation is that, compared to a single RCM image input, a 3D RCM image stack containing multiple adjacent slices provides a more accurate basis for learning and virtual staining inference due to the additional input information provided by the 3D spatial structure.

Using the presented virtual staining framework, we showed good concordance between virtual histology and common histologic features in the stratum spinosum, dermal-epidermal junction, and superficial dermis, areas of skin most commonly involved in pathological conditions. Virtually-stained RCM images of BCC show analogous histological features including nodules of basaloid cells with peripheral palisading, mucin, and retraction artifact. These same features are used to diagnose BCC from skin biopsies by pathologists using H&E histology. In addition to these, we also demonstrated that the virtual staining network successfully inferred pigmented melanocytes in benign melanocytic nevi (see Fig. S6). The success of our virtual staining results can be due to the connection between certain histological structures and the corresponding RCM signals, caused by e.g., the unique reflectance/refraction properties of collagen. For example, fibrotic collagen that is present in multiple types of skin cancer is highly reflective, leading to bright RCM signals. Therefore, the presence of fibrotic collagen creates a bridge that enables the successful transformation of RCM signals into virtual staining of histological features of BCC.

While our results demonstrate the proof of concept in obtaining histology quality images in vivo without the need for invasive biopsies, several limitations remain for future work. First, we had a limited volume of training data which was primarily composed of nodular BCC, which contained round nodules. When applied to another type of BCC from the blind testing set containing infiltrative, strand-like tumor islands of BCC with focal keratinization, it resulted in a form of an artifact composed of dark blue/purple streaks of basaloid cells similar to the cords/strands seen in the microscopic image of frozen section histology from this sample, but with lower resolution (see Fig. S4). The bias of the training set towards nodular BCC may have hampered the generalization performance of the network. In order to address this issue, additional data on different types of BCC would be needed for training the network to recognize differences in the nuclear structure of BCC subtypes.

Another limitation of our virtual histology framework is that not all nuclei were placed with perfect fidelity in the transformed, virtually stained images. In our quantitative analysis for the prediction of nuclei, there remained a positional misalignment between the network inputs and the corresponding ground truth images. This resulted in relatively imprecise learning of the image-to-image transformation for virtual staining and therefore can be thought of as “weakly paired” supervision^44^. To mitigate this misalignment error in the training image acquisition (time-lapsed RCM imaging process), one can reduce the number of RCM images in a stack in order to decrease the time interval between successive RCM stacks. This may help capture more continuous 3D training image sequences to improve the initial registration of the ground truth images with respect to the input images. We can also further improve our learning-based image registration algorithm, detailed in Fig. 2d, to be able to process volumetric spatial information in 3D RCM image stacks, helping to reduce axial misalignment errors due to e.g., sample deformation during the staining process, which can cause translation and tilting of the target image plane.

Furthermore, distinct nuclei in virtually stained RCM images of BCC tumor islands did not show exactly the same placement, size, and patterns as with ex vivo ground truth acetic acid staining and standard histology results; see Fig. 6, third row, Fig. 7 and Fig. S4. There are a few possible reasons for the disparate images in individual basaloid keratinocytes of BCC on a cellular level. First, individual cells of BCC are derived from the basal cell layer (or progenitor layer) of the epidermis, thus cells of BCC resemble “basaloid cells”. Specific histologic features of basaloid cells (including BCC) have a small size, a high nuclear to cytoplasmic ratio, a rounded appearance, with less keratinization (resulting in a blue/purple appearance of cells) when compared to keratinocytes of the spinous and granular layer which are larger, flatter, with a lower nuclear to cytoplasmic ratio, and more keratinization (resulting in a pink appearance to cells) than basaloid cells. Tumor islands in BCC are composed of these smaller cells, making them more densely packed with cells than those of normal skin^45^. Second, raw RCM images of BCC tumor islands (both in vivo and ex vivo) lack clearly defined cell borders inside BCC tumor islands. A likely reason for this may be the presence of mucin, which absorbs the penetrated light and reduces the reflectance of peripheral spatial features that are used for neural network inference. For instance, mucin is observed in Fig. 6i, l, as well as Fig. S5i as indistinct white to pale-blue patches without cellular features in the center of (or surrounding) tumor islands. Coincidentally, this non-distinct pale blue patch is how mucin appears in histochemically-stained H&E sections and often requires special staining with Alcian Blue and Colloidal Iron staining to become microscopically visible^46^. Third, as light from the RCM penetrates deeper into the tissue, image resolution decreases due to a lower signal-to-noise ratio (SNR). The above-mentioned factors may affect the performance of the neural network to appropriately assign individual cell nuclei within BCC tumor islands.

Overall, the described virtual histology approach can allow diagnosticians to see the overall histological features, and obtain in vivo “gestalt diagnosis”, as pathologists do when they examine histology slides at low magnification^47^. We also collected ground truth histology from the same specimen used for RCM imaging, as reported in Fig. 7j, k and Fig. S1, and showed that virtual and ground truth histology images share similar features. Due to the series of complicated and destructive operations required for biopsy and H&E histochemical staining, we were naturally unable to compare identical regions of in vivo and ex vivo RCM processed H&E histology. Overall, our results show that the virtual staining networks can reconstruct BCC nodules and melanocytes within nevi appropriately with features and color contrast commonly seen in histologically-stained microscopy sections.

Further investigation is required to understand how virtual histology affects diagnostic accuracy, sensitivity, and specificity when compared to the grayscale contrast of RCM images. Moreover, larger datasets and clinical studies are needed to further evaluate the utility of the virtual histology algorithm. Our training dataset was predominantly normal skin samples and nodular and superficial types of BCC. In future work, we will collect more BCC and additional BCC-subtype data to assess the network’s ability to detect cell nuclei inside basal cell tumor islands. Since the presence of multiple types of immune cell infiltration (i.e., tumor-infiltrating lymphocytes) and nonimmune changes to the stroma/extracellular matrix (i.e., thick, fibrotic collagen) in the tumor microenvironment is critical for diagnosis, prognosis, and response to immunotherapy^48–50^, increasing the volume and diversity of the training BCC data will be able to more accurately represent these changes within the tumor microenvironment, which will be critical for the use of RCM-based virtual histology to diagnose skin cancers and lend prognostic information. For this aim, future clinical studies should address whether our approach improves the diagnostic interpretation of skin conditions by expert RCM users, and reduces the amount of advanced training required for novice RCM users. Furthermore, the ability to switch between the original grayscale and pseudo-H&E virtual staining mode in real-time may further improve the diagnostic capabilities of in vivo RCM. Finally, if image stacks acquired at successive depths in the horizontal plane are reconstructed to produce virtually stained volumetric data, images can also be examined in the vertical plane in a similar fashion to traditional skin histology.

In addition to these, we can also collect training data from other imaging modalities to further advance the presented 3D virtual staining framework. For example, multimodal ex vivo reflectance and fluorescence confocal microscopy systems are compatible with conventional nuclear stains, such as acridine orange, and other fluorescent stains to better illuminate different features of the tumor microenvironment, including fibrotic collagen, inflammatory cells, and mucin. Multiphoton microscopy can also illuminate other endogenous structures, providing more detailed information regarding the organization of the collagen fibrils. Recent studies have also used deep learning to infer fluorescence and nonlinear contrast from the texture and morphology of RCM images by using multiphoton microscopy images as ground truth^51,52^. Additional training data from these different microscopy modalities and image contrast mechanisms can potentially be used to further improve our 3D virtual staining approach.

All in all, we reported deep learning-enabled in vivo virtual histology to transform RCM images into virtually-stained images for normal skin, BCC, and melanocytic nevi. Future studies will evaluate the utility of our approach across multiple types of skin neoplasms and other noninvasive imaging modalities towards the goal of optical biopsy enhancement for noninvasive skin diagnosis.

## Materials and methods

### In vivo RCM image acquisition

Following informed consent (Advarra IRB, Pro00037282), 8 patients had RCM images captured during regularly scheduled visits. RCM images were captured with the VivaScope 1500 System (Caliber I.D., Rochester, NY), by a board-certified dermatologist trained in RCM imaging and analysis. RCM imaging was performed through an objective lens-to-skin contact device that consists of a disposable optically clear window. The window was applied to the skin over a drop of mineral oil and used throughout the imaging procedure. The adhesive window was attached to the skin with a medical-grade adhesive (3 M Inc., St. Paul, MN). Ultrasound gel (Aquasonic 100, Parker Laboratories, Inc.) was used as an immersion fluid, between the window and the objective lens. Approximately three RCM mosaic scans and two z-stacks were captured stepwise at 1.52 or 4.56 µm increments of both normal skin and skin lesions suspicious for BCC. Large movements by the patient can cause changes in the axial position of the sample while acquiring RCM images, resulting in misaligned and motion-blurred mosaic and z-stack images. If this occurs, it is standard practice for the medical personnel acquiring RCM images to detect the anomaly and reacquire the image set. Nevertheless, if the movements are relatively mild and the sharpness of the RCM images is retained, these images can still be interpreted and used for network inference after successfully applying image stack registration (see the “Image preprocessing and registration” subsection in Materials and Methods for more details). Upon completion of RCM imaging, patients were managed as per standard-of-care practices. In several cases, skin lesions that were imaged in vivo were subsequently biopsied or excised using standard techniques and the excised tissue was subjected to ex vivo RCM imaging and/or diagnostic tissue biopsy. Tissue diagnosis was confirmed by a board-certified dermatopathologist.

The final in vivo blind testing dataset that we used to present the in vivo results reported in this paper was composed of 979 896 × 896 RCM images collected in vivo without any acetic acid-stained ground truth. Histopathologic confirmation was obtained on all skin lesions/tumors but was not provided on in vivo RCM images of normal skin.

### Skin tissue sample preparation for ex vivo RCM imaging

Discarded skin tissue specimens from Mohs surgery tissue blocks (from 36 patients) with and without residual BCC tumor were retrieved for ex vivo RCM imaging with IRB exemption determination (Quorum/Advarra, QR#: 33993). Frozen blocks were thawed, and the specimens were thoroughly rinsed in normal saline. Small samples of intact skin stratum corneum, epidermis and superficial dermis were trimmed from tissue specimens. The skin sample length and width varied depending on the size of the discarded Mohs specimen. The adipose and subcutaneous tissue was trimmed from the superficial skin layers, such that skin samples from the stratum corneum to the superficial dermis were ~2 mm thick. The trimmed skin samples were placed flat onto an optically clear polycarbonate imaging window with the stratum corneum side down and placed in a tissue block made from 4% agarose (Agarose LE, Benchmark Scientific). The agarose solution was brought to a boiling point and ~0.1–0.3 mL was pipetted over the trimmed skin sample and imaging window until that the entire sample was covered by the agarose solution. About 10 min was given for the agarose solution to cool to room temperature, hardening into a malleable mold that encapsulated the skin tissue sample flat against the imaging window. A 2 mm curette was used to channel a small opening in the agarose mold to access the center of the skin tissue sample while the perimeter of the sample remained embedded in the agarose mold.

### Ex vivo RCM image acquisition of tissue blocks

The imaging window with the agarose molded skin tissue was attached to the RCM device (VivaScope 1500, Caliber I.D., Rochester, NY), which operates at a frame rate of 9 frames/sec. Ultrasound gel (Aquasonic 100, Parker Laboratories, Inc.) was used as an immersion fluid, between the window and the objective lens. The optical head of the RCM device was inverted. Image z-stacks containing 40 images each were captured stepwise with 1.52 µm increments to a total depth of 60.8 µm. About 10–20 consecutive image stacks were captured in a continuous time-lapse fashion over the same tissue area. Areas with features of interest (e.g., epidermis, dermal-epidermal junction, superficial dermis, etc.) were selected before imaging. The first image stack captured RCM images of label-free skin tissue. After completion of the first image stack, 1–2 drops of 50% acetic acid solution (Fisher Scientific) were added to a small opening in the agarose mold with access to the center of the skin tissue sample. While 5% acetic acid is sufficient to stain nuclei of normal skin tissue, a higher concentration was required to penetrate mucin that often surrounds islands of BCC tumor, and thus a standard 50% solution was added to all tissue. RCM time-lapse imaging continued until acetic acid penetrated the area of interest and stained cell nuclei throughout the depth of the image stack. Before and after time-lapse imaging, RCM mosaics (Vivablocks) of the skin tissue sample were also captured at one or several depths. After ex vivo RCM imaging, samples were either fixed in 10% neutral buffered formalin (Thermo Fisher Scientific, Waltham, MA) for histopathology or safely discarded.

The final ex vivo training, validation, and testing datasets that were used to train the deep network and perform quantitative analysis of its blind inference results were composed of 1185, 137, and 199 896 × 896-pixel ex vivo RCM images of unstained skin lesions and their corresponding acetic acid-stained ground truth, which were obtained from 26, 4, and 6 patients, respectively.

### Image preprocessing and registration

Accurate alignment of the training image pairs is of critical importance for the virtual staining deep neural network to learn the correct structural feature mapping from the unstained tissue images to their stained counterparts. The principle of our image registration method relies on the spatial and temporal consistency of the time-lapse volumetric image stack captured using RCM during the staining process of the ex vivo tissue samples. In other words, the raw data cover essentially 4-dimensional space, where the three dimensions represent the volumetric images of the tissue and the fourth dimension (time) records the whole staining process of the tissue, i.e., from the unstained state to the stained state, as a function of time.

Figure 2a provides an overview of the image registration workflow. The first part of our registration process starts with performing an “initial registration” to achieve coarsely registered image pairs, which includes two sub-steps as depicted in Fig. 2b. In sub-step (1) of the initial registration, we manually selected a certain depth of the time-lapse volumetric image stack at hand, and iteratively applied a pyramid elastic registration algorithm^25,26,30,32,53^ (see Supplementary Note 1 for details) to each of the image pairs that are at this depth, but captured at successive time points. For this, we used an image sequence where all the images are located at the same depth and aligned throughout the staining process. In sub-step (2) of the initial registration, we manually inspected the images in this aligned image sequence and picked two images that have 0 and 100% nuclei stained, i.e., referring to “before staining” and “after staining” phases, respectively. We found the corresponding z-stacks that these two picked images belong to and performed a stack registration based on the same elastic registration algorithm used in sub-step (1). As a result of this initial registration process, all the images in these two stacks were roughly aligned with each other, by and large eliminating the large-scale elastic deformations that occurred during the imaging and staining process, forming the initially-registered input-target image pairs.

At this stage, it is noteworthy that small shifts and distortions between the two sets of initially-registered images can still exist and lead to errors during the learning process. To mitigate this, we further aligned these image pairs at a sub-pixel level through the second part of our registration process. In this part, the coarsely registered image pairs were individually fed into a convolutional neural network *A*, whose structure is similar to the generator network reported in Fig. 3 except that the number of channels and downsampling operations are fewer, and the first few 3D convolutions are replaced with 2D convolutions (see the “Network architecture and training schedule” subsection in Materials and Methods for details). Then, a soft training of network *A* using all these images is utilized to transform the input images to visually resemble the sought target. The aim of this method is to build an initial bridge between the input and target images to facilitate their accurate alignment. Using the pyramid elastic registration method (see Supplementary Note 1 for details), we aligned the target images against the output of network *A*, thus achieving more accurate spatial correspondence between the unstained input and the corresponding target images; we term this step as the “first fine registration”. Note that all the elastic registration algorithms mentioned till now perform spatial transformation based on a displacement vector field (DVF) of the image pair, which is calculated through the multi-scale correlation between the two images that form a pair; see Fig. 2c.

Despite its utility, the calculation of multi-scale correlation can frequently produce abnormal values on DVFs, which result in unsmooth distortions in the registered images from time to time. To mitigate this problem, we applied another round of soft training of a separate network *A*′ (that is similar to *A*) and a second fine registration step to further improve the registration accuracy. Unlike the first fine registration, this second fine registration step was performed based on the DVF generated by a learning-based algorithm^54^, where a deep convolutional neural network *B* is trained to learn the smooth, accurate DVF between two input images. The training details of this network *B* are reported in Fig. 2d. In the training phase, the network *B* is fed with the cropped patches of the output of network *A*′*,* i.e., *I*_f_, along with the roughly registered target image patches, *I*_m_, and generates a predicted DVF *ϕ* that indicates the pixel-wise transformation from *I*_m_ to *I*_f_, such that *I*_m_ serves as “moving” patches and *I*_f_ serves as “fixed” patches. Then, *I*_m_ is spatially deformed using *ϕ* so that the predicted registered target patches, *I*_r_, are produced. To create the data with smooth and accurate spatial transformations, serving as ground truth for training *B*, we performed the previous pyramid elastic registration (based on multi-scale correlation, see Supplementary Note 1 for details) once again using only ~10% of our roughly registered image pairs (i.e., output images of *A*′ and their roughly registered targets). During this process, we fine-tuned the pyramid elastic registration algorithm to obtain optimal spatial transformations so that we achieved the accurately registered target patches *I*_r,gt_ and the corresponding DVFs *ϕ*_gt_. Using these *I*_r,gt_ and *ϕ*_gt_ with their corresponding *I*_m_ and *I*_f_, we formed a training set and performed the supervised training of the network *B*, where the loss function was selected to minimize the difference of both (*I*_*r*_-*I*_r,gt_) and (*ϕ*-*ϕ*_gt_) using mean square error loss, and the total variation (TV) of *ϕ*. Once the network *B* was successfully trained and used to perform inference across the entire image dataset, the target images were much more accurately aligned with the output of network *A*′, eliminating various registration artifacts. Finally, through this approach, we generated the registered acetic acid-stained target images that are aligned accurately against the unstained/label-free input RCM images, making it ready for training the acetic acid virtual staining network (VS_AA_), which will be detailed next.

Apart from these image preprocessing and registration procedures for network training, we also applied the same (pyramid elastic) stack registration algorithm to the RCM image stacks used for inference, e.g., in vivo blind testing images. This is necessary because even mild motion of patients that might occur during the image capture usually brings strong misalignment and deformation to different layers of the image stack. If the image stack registration is not performed here, this misalignment will cause our network inference to fail. Our image stack registration workflow is able to successfully correct a lateral shift of up to ~20 μm within a given field of view with sub-pixel accuracy. Supplementary Videos 1–4 are provided to exemplify the success of our image stack registration algorithm, correcting the shifts and deformations caused by undesired motion.

### Generative model and loss functions

In this work, we utilized a pix2pix GAN framework^55^ as our generative model of acetic acid virtual staining network (VS_AA_), which includes the training of (1) a generator network for learning the statistical transformation between the unstained input RCM image stacks and the corresponding acetic acid-stained tissue images and (2) a discriminator network for learning how to discriminate between a true RCM image of an actual acetic acid-stained skin tissue and the generator network’s output, i.e., the corresponding virtually stained (acetic acid) tissue image. The merit of using this pix2pix GAN framework stems from two aspects. First, it retains the structural distance penalty in a regular deep convolutional network, so that the predicted virtually stained tissue images can converge to be similar with their corresponding ground truth in overall structural features. Second, as a GAN framework, it introduces the competence mechanism by training the two aforementioned networks in parallel. Due to the continuous enhancement of the discrimination ability of the discriminator network during the training process, the generator must also continuously generate more realistic images to deceive the discriminator, which gradually impels the feature distribution of the high-frequency details of the generated images to conform to the target image domain. Ultimately, the desired result of this training process is a generator, which transforms an unstained input RCM image stack into an acetic acid virtually stained tissue image that is indistinguishable from the actual acetic acid-stained RCM image of the same sample at the corresponding depth within the tissue. To achieve this, following the GAN scheme introduced above, we devised the loss functions of the generator and discriminator networks as follows:

1 $$\begin{array}{ll} {{{\mathcal{L}}}}_{{{{\mathrm{generator}}}}} = {{{\mathcal{L}}}}_{{{{\mathrm{structural}}}}}\left\{ {I_{{{{\mathrm{target}}}}},G(I_{{{{\mathrm{input}}}}\_{{{\mathrm{stack}}}}})} \right\}\\ \qquad\qquad\quad+\, \alpha \times TV\left\{ {G(I_{{{{\mathrm{input}}}}\_{{{\mathrm{stack}}}}})} \right\}\\ \qquad\qquad\quad+\, \lambda \times \left( {1 - D\left( {G(I_{{{{\mathrm{input}}}}\_{{{\mathrm{stack}}}}})} \right)} \right)^2 \end{array}$$

2 $$\begin{array}{*{20}{c}} {{{{\mathcal{L}}}}_{{{{\mathrm{discriminator}}}}} = D\left( {G(I_{{{{\mathrm{input}}}}\_{{{\mathrm{stack}}}}})} \right)^2 \;+\; \left( {1 - D\left( {I_{{{{\mathrm{target}}}}}} \right)} \right)^2} \end{array}$$

where $G( \cdot )$ represents the output of the generator network, $D( \cdot )$ represents the output probabilistic score of the discriminator network, $I_{{{{\mathrm{target}}}}}$ denotes the image of the actual acetic acid-stained tissue used as ground truth, *I*_input_stack_ denotes the input RCM image stack (unstained). The generator loss function Eq. (1) aims to balance the pixel-wise structural error of the generator network output image with respect to its ground truth target, the total variation (TV) of the output image, and the discriminator network’s prediction of the generator network’s output, using the regularization coefficients (α, λ) that are empirically set as (0.02, 15). Specifically, the structural error term ${{{\mathcal{L}}}}_{{{{\mathrm{structural}}}}}$ takes a form of the reversed Huber (or “BerHu”) error, which blends the traditional mean squared error and mean absolute error using a certain threshold as the boundary. The reversed Huber error between 2D images *a* and *b* is defined as:

3 $${{{\mathcal{L}}}}_{{{{\mathrm{BerHu}}}}}\left\{ {a,b} \right\} = \sum\limits_{\mathop {m,n}\limits_{|a(m,n) - b(m,n)| \le \delta } } \left| {a\left( {m,n} \right) - b\left( {m,\,n} \right)} \right|\,+\sum\limits_{\mathop {m,n}\limits_{|a(m,n) - b(m,n)| > \delta } } \frac{{\left| {a\left( {m,n} \right) - b\left( {m,\,n} \right)} \right|^2 + \delta ^2}}{{2\delta }}$$

where *m*,*n* are the coordinates on the images, and *δ* is a threshold hyperparameter that is empirically set as 20% of the standard deviation of the normalized ground truth image $z_{{{{\mathrm{target}}}}}$. The third term of Eq. (1) penalizes the generator to produce outputs that are more realistic to the discriminator by maximizing the discriminator’s response to be 1 (real, like an actual acetic acid-stained tissue image), which increase the authenticity of the generated images. The discriminator loss function Eq. (2) attempts to achieve the correct classification between the network’s output and its ground truth by minimizing the score of the generated image to be 0 (classified to be a virtually stained tissue image) and maximizing the score of the actual acetic acid-stained tissue image to be 1 (real, classified to be actual/real acetic acid-stained tissue image). Within this adversarial learning scheme^56^, we also applied spectral normalization^57^ in the implementation of the discriminator network to improve its training stability.

### Network architecture and training schedule

For the generator network, as shown in Fig. 3a, we employed an attention U-Net structure (encoder-decoder with skip connections and attention gates)^58,59^ to learn the 3D transformation from the label-free unstained RCM image stack to the acetic acid virtually stained tissue image, which was adapted to work on 3D input distributions, matching our input RCM image stacks. For each sample, a stack of 7 RCM images (unstained) adjacent in depth and with an axial step size of 1.52 μm are used as the network input and encoded in the depth dimension of the network, and the U-Net generates a single virtually stained tissue image that is corresponding to the central plane of the image stack. In other words, the output image is at the same level as the fourth image in the input stack. In the U-Net structure, there is a downsampling path and a symmetric upsampling path. In the downsampling path, there are five convolution–downsampling blocks, each consisting of (1) three 3 × 3 successive 2D convolutional layers with batch normalization layers and leaky rectified linear unit (leaky ReLU, with a slope of 0.2) in between to extract and encode spatial features and (2) one 2 × 2 2D average pooling layer with a stride of 2 × 2 to perform a 2x downsampling. Note that rather than using 2D convolution, the first block uses three 3D convolutional layers with a kernel size of 3 × 3 × 3 and without padding in the depth dimension, which shrinks (after three layers) the depth size of the input tensor from 7 to 1, resulting in 2D outputs that are consistent with the following convolutional operations of the U-Net structure. Also, there is a residual connection communicating the first and last tensor in each block with an addition operation. Following the downsampling path, the upsampling path has five corresponding convolution–upsampling blocks. The input of each block is a channel dimension concatenation of the output tensor of the previous block in the upsampling path and the attention gated output tensor at the corresponding level in the downsampling path, which creates skip connections between the upsampling path and downsampling path. It is worth noting that to alleviate irrelevant spatial information propagated in the simple skip connection of the U-Net, we also employed soft attention gate blocks in each skip connection, including a few convolutional layers and a sigmoid operation to calculate the activation weight maps, such that the feature maps from the downsampling encoder path are pixel-wise multiplicatively weighted and propagated to the upsampling decoder path. The structure of the upsampling block is quite similar to the downsampling path, except that (1) the pooling layers are replaced by 2x bilinear upsampling layers and (2) there is no residual connection.

As depicted in Fig. 3b, the discriminator is a convolutional neural network that consists of five successive convolutional blocks. Each block is composed of one 3 × 3 2D convolutional layer with a stride of 1 × 1, one 2 × 2 2D convolutional layer with a stride of 2 × 2 to perform 2× downsampling and leaky ReLU layers after each convolutional layer. After the last convolutional block, an average pooling layer flattens the output tensor to 1 × 1 but keeps the channel dimension, subsequently fed into a two-layer fully connected block of size 1024 × 1024 and 1024 × 1. The final output represents the discriminator probabilistic score, which falls within (0, 1), where 0 represents a false and 1 represents a true label.

During the training of this GAN framework, we randomly cropped the input image stacks and the registered target images to patch sizes of 256 × 256 × 7 and 256 × 256, respectively and used a batch size of 12. Before feeding the input images we also applied data augmentation, including random image rotation, flipping, and mild elastic deformations^60^. The learnable parameters were updated through the training stage of the deep network using an Adam optimizer^61^ with a learning rate of 1 × 10^−4^ for the generator network and 1 × 10^−5^ for the discriminator network. Also, at the beginning of the training, for each iteration of the discriminator, there are 12 iterations of the generator network, to avoid the mode collapse, following potential overfitting of the discriminator network to the targets. As the training evolves, the number of iterations ($t_{G{{{\mathrm{per}}}}D}$) of the generator network for each iteration of the discriminator network linearly decreases, which is given by

4 $$\begin{array}{*{20}{c}} {t_{G{{{\mathrm{per}}}}D} = {\mathrm{max}}\left( {3,\bigg\lfloor\,12 - 0.25\big\lfloor\frac{{t_D}}{{1000}}}\big\rfloor\bigg\rfloor \right)} \end{array}$$

where *t*_*D*_ denotes the total number of iterations of the discriminator, $\left\lfloor \cdot \right\rfloor$ represents the ceiling functions. Usually, the *t*_*D*_ is expected to be ~40,000 iterations when the generator network converges. A typical plot of the loss functions during the GAN training is shown in Fig. S8.

### H&E virtual staining

For the pseudo-H&E virtual staining of the actual and virtual acetic acid-stained tissue images in this work, we modified an earlier approach^62^, where epi-fluorescence images were used to synthesize pseudo-color images with H&E contrast. The principle of our pseudo-H&E virtual staining relies on the characteristics of H&E staining that the nucleus and cytoplasm are stained with blue and pink, respectively. In our work, an unstained input image collected by RCM (*I*_input_) and its corresponding actual acetic acid-stained tissue image (*I*_target_) are subtracted in pixel intensities to extract the foreground component $I_{{{{\mathrm{foreground}}}}}$ that mainly contains the nuclear features:

5 $$\begin{array}{*{20}{c}} {I_{{{{\mathrm{foreground}}}},{{{\mathrm{target}}}}} = max\left( {1.2 \times I_{{{{\mathrm{target}}}}} - 0.8 \times I_{{{{\mathrm{input}}}}},0} \right)} \end{array}$$

Note that *I*_target_ and *I*_input_ are initially normalized to (0, 1), and all the operations in Eq. (5) are pixel-wise performed on the 2D images. The selection of the coefficients 1.2 and 0.8 here is empirical. The background component that contains other spatial features including cytoplasm is defined by simply using the unstained input images *I*_input_. Following this separation of the foreground and background components, a pseudo-H&E acetic acid-stained tissue image ${{{\boldsymbol{I}}}}_{{{{\mathbf{analytical}}}} - {{{\mathbf{HE}}}},{{{\mathbf{target}}}}}$ is analytically computed by colorizing and blending these two components based on a rendering approach, which models transillumination absorption using the Beer–Lambert law^62^:

6 $$\begin{array}{*{20}{c}} {{{\boldsymbol{I}}}}_{{{{\mathbf{analytical}}}} - {{{\mathbf{HE}}}},{{{\mathbf{target}}}}} = exp\left( { - {\it{\it{\upbeta} }}_{{{{\mathbf{hematoxylin}}}}}\;I_{{{{\mathrm{foreground}}}},{{{\mathrm{target}}}}}} \right)\\ \qquad\quad{exp}\left( { - {\it{\upbeta} }_{{{{\mathbf{eosin}}}}}\;I_{{{{\mathrm{input}}}}}} \right) \end{array}$$

where ***β***_**hematoxykin**_ and ***β***_**eosin**_ are the three-element weight vector corresponding to R, G, and B channels that helps to mimic the real color of hematoxylin and eosin, respectively. In our work, the values of the elements in ***β***_**hematoxykin**_ and ***β***_**eosin**_ are empirically chosen as [0.84, 1.2, 0.36]^T^ and [0.2, 2, 0.8]^T^, respectively. Similarly, a pseudo-H&E acetic acid virtually stained tissue image ${{{\boldsymbol{I}}}}_{{{{\mathbf{analytical}}}} - {{{\mathbf{HE}}}},{{{\mathbf{output}}}}}$ can also be computed by replacing *I*_target_ with an acetic acid virtually stained tissue image *I*_output_ in Eq. (5).

This analytical approach (Eq. 6) works well on most of the actual and virtual acetic acid-stained tissue images to create H&E color contrast. However, when it comes to the images that contain melanocytes, whose H&E stain produces dark brown, this algorithm fails to generate the correct color at the position of these melanocytes. Considering that the brown color (representing melanin) would not be possible to generate through a pixel-wise linear combination of the images *I*_input_ and *I*_target_ or *I*_output_, we introduced a learning-based approach to perform the correct pseudo-H&E virtual staining (VS_HE_), which can incorporate inpainting of the missing brown features by using the spatial information content of the images. For training purposes, we performed manual labeling of melanocytes to create training data for this learning-based approach. In order to reduce the labor of this manual labeling, we first estimated the initial distribution of melanin in a certain field of view through an empirical formula:

7 $$\begin{array}{*{20}{c}} {I_{{{{\mathrm{melanin}}}}} = \left\{ {\begin{array}{*{20}{c}} {I_{{{{\mathrm{input}}}}}}, & {\,{\mathrm{where}}\;\,I_{{{{\mathrm{target}}}}} \cdot I_{{{{\mathrm{input}}}}} \,>\, I_{{{{\mathrm{th}}}}}} \\ 0, & {\,{\mathrm{otherwise}}} \end{array}} \right.} \end{array}$$

where $\cdot$ denotes pixel-wise multiplication, and *I*_th_ represents a threshold that is selected as 0.2 based on empirical evidence. The constitution of this formula is based on the observation that melanin has strong reflectance in both the unstained/label-free and actual acetic acid-stained tissue RCM images, namely *I*_input_ and *I*_target_, respectively. Then, these initial estimations are further cleaned up through a manual labeling process performed with the assistance of a board-certified dermatopathologist, resulting in $I_{{{{\mathrm{melanin}}}},{{{\mathrm{labeled}}}}}$. This manual labeling process as part of our training forms the core task that will be learned and executed by our learning-based scheme. Similar to Eq. (6) but with one more term added, the corrected pseudo-H&E virtual staining results for the actual acetic acid-stained tissue images ${{{\tilde{\boldsymbol I}}}}_{{{{\mathbf{analytical}}}} - {{{\mathbf{HE}}}},{{{\mathbf{target}}}}}$ can be computed as:

8 $$\begin{array}{*{20}{c}} {{{\tilde{\boldsymbol I}}}}_{{{{\mathbf{analytical}}}} - {{{\mathbf{HE}}}},{{{\mathbf{target}}}}} = exp\left( { - {\it{\upbeta }}_{{{{\mathbf{hematoxylin}}}}}\;I_{{{{\mathrm{foreground}}}},{{{\mathrm{target}}}}}} \right)\\{exp}\left( { - {\it{\upbeta }}_{{{{\mathbf{eosin}}}}}\;I_{{{{\mathrm{input}}}}}} \right)exp\left( { - {\it{\upbeta }}_{{{{\mathbf{brown}}}}}\;I_{{{{\mathrm{melanin}}}},{{{\mathrm{labeled}}}}}} \right) \end{array}$$

where the value of ***β***_**brown**_ is empirically chosen as $\left[ {0.12,\,0.24,\,0.28} \right]^T$ in order to correctly render the brown color of the melanin. Using Eq. (8), we obtained the ground truth images for the learning-based virtual staining approach to perform the corrected pseudo-H&E virtual staining. Using the ex vivo training set, we trained the pseudo-H&E virtual staining network VS_HE_ to transform the distribution of the input and actual acetic acid-stained tissue images, i.e., *I*_input_ and *I*_target_, into ${{{\tilde{\boldsymbol I}}}}_{{{{\mathbf{analytical}}}} - {{{\mathbf{HE}}}},{{{\mathbf{target}}}}}$. The architecture of the network VS_HE_ is identical to the ones used in our registration process, except for that the input and output of the network VS_HE_ have two and three channels, respectively. Once the training is finished, we used the resulting network VS_HE_ to perform pseudo-H&E virtual staining of our previously generated acetic acid virtually stained tissue images *I*_output_ in the testing set. The network VS_HE_ took *I*_output_ along with input images *I*_input_ to generate pseudo-H&E virtually stained tissue images ${{{\tilde{\boldsymbol I}}}}_{{{{\mathbf{VS}}}} - {{{\mathbf{HE}}}},{{{\mathbf{output}}}}}$ with the correct color for melanin:

9 $$\begin{array}{*{20}{c}} {{{{\tilde{\boldsymbol I}}}}_{{{{\mathbf{VS}}}} - {{{\mathbf{HE}}}},{{{\mathbf{output}}}}} = {{{\mathrm{VS}}}}_{{{{\mathrm{HE}}}}}\left( {I_{{{{\mathrm{output}}}}},\,I_{{{{\mathrm{input}}}}}} \right)} \end{array}$$

We used Eq. (9) to create all the pseudo-H&E virtually stained tissue images reported in our main text. To exemplify the effectiveness of this learning-based pseudo-H&E virtual staining approach, in Fig. S9 we also present a comparison between the pseudo-H&E virtual staining results against their counterparts generated by Eq. (8) using a few examples on the testing test, which demonstrates a decent correspondence between the two approaches.

### Quantitative morphological analysis of virtual staining results

CellProfiler^63^ was used to conduct morphological analysis of our results. After loading our actual acetic acid-stained tissue images and virtually stained (acetic acid) tissue images using CellProfiler, we performed cell segmentation and profile measurement to quantitatively evaluate the quality of our predicted images when compared with the corresponding ground truth images. In CellProfiler, the typical diameter of objects to detect (i.e., nuclei) was set to 10–25 pixel units and objects that were outside the diameter range or touching the border of each image were discarded. We applied an adaptive thresholding strategy using minimum cross-entropy with a smoothing scale of 6 and a correction factor of 1.05. The size of the adaptive window was set to 50. “Shape” and “Propagate” methods were selected to distinguish the clumped objects and draw dividing lines between clumped objects, respectively. Following this step, we then introduced the function module “IdentifyPrimaryObjects” to segment the nuclei in a slice-by-slice manner. Accordingly, we achieved well-segmented nuclei images containing positional and morphological information associated with each detected nuclear object.

For the analysis of nuclear prediction performance of our model, we first employed the function module “ExpandOrShrinkObjects” to slightly expand the detected nuclei by e.g., 4 pixels (~2 µm), so that the image registration and nuclei tracking-related issues across different sets of images can be mitigated. Then we used the function module “RelateObjects” to assign a relationship between the objects of virtually stained nuclei and actual acetic acid-stained ground truth, and used “FilterObjects” to only retain the virtually stained nuclei objects that present overlap with their acetic acid-stained ground truth, which were marked as true positives (TP). Similarly, false positives (FP) and false negatives (FN) were marked based on the virtually stained nuclei objects that have no overlapping with their ground truth, and the actual acetic acid-stained nuclei objects that have no overlap with the corresponding virtually stained nuclei objects, respectively. Note that in this case, we do not have true negative (TN) calculated since we cannot define a nuclear object that does not exist in both the virtually-stained and ground truth images. Next, we counted the numbers of TP, FP, and FN events, which were denoted as $n_{{\mathrm{TP}}}$, $n_{{\mathrm{FP}}}$, and $n_{{\mathrm{FN}}}$, respectively, and accordingly computed the Sensitivity and Precision values, defined as:

10 $$\begin{array}{*{20}{c}} {{\mathrm{Sensitivity}} = \frac{{n_{{\mathrm{TP}}}}}{{n_{{\mathrm{TP}}} \;+\; n_{{\mathrm{FN}}}}}} \end{array}$$

11 $$\begin{array}{*{20}{c}} {{\mathrm{Precision}} = \frac{{n_{{\mathrm{TP}}}}}{{n_{{\mathrm{TP}}} \;+\; n_{{\mathrm{FP}}}}}} \end{array}$$

For the nuclear morphological analysis, we utilized the function module “MeasureObjectSizeShape” to compute the nuclei area (“AreaShape_Area”, the number of pixels in one nucleus), compactness (“AreaShape_Compactness”, the mean squared distance of the nucleus’s pixels from the centroid divided by the area of the nucleus), and eccentricity (“AreaShape_Eccentricity”, the ratio of the distance between the foci of the effective ellipse that has the same second-moments as the segmented region and its major axis length). The “MeasureObjectIntensity” module was employed afterward to compute the nuclei reflectance (“Intensity_IntegratedIntensity_Cell”, the sum of the pixel intensities within a nucleus). We finally utilized the function module “MeasureTexture” to compute the contrast of the field of view (“Texture_Contrast_Cell”, a measure of local variation in an image). For image similarity analysis, we calculated the Pearson Correlation Coefficient (PCC) for each image pair of the virtual histology results and the corresponding ground truth image based on the following formula:

12 $$\begin{array}{*{20}{c}} {PCC = \frac{{{\sum} {(I_{{{{\mathrm{output}}}}} \;- \;{E}(I_{{{{\mathrm{output}}}}}))(I_{{{{\mathrm{target}}}}} \;- \;{E}(I_{{{{\mathrm{target}}}}}))} }}{{\sqrt {{\sum} {(I_{{{{\mathrm{output}}}}} \;-\; E(I_{{{{\mathrm{output}}}}}))^2} } \sqrt {{\sum} {(I_{{{{\mathrm{target}}}}} \;-\;{E}(I_{{{{\mathrm{target}}}}}))^2} } }}} \end{array}$$

where *I*_output_ and *I*_target_ represent the predicted (virtually-stained) and ground truth images, respectively, and $E( \cdot )$ denotes the mean value calculation. For all the violin plots presented above, we used the violin plot function in the Seaborn Python library^64^ to visualize the conformance between the prediction and ground truth images.

### Network implementation details

The deep neural networks used in this work were implemented and trained using Python (v3.6.5) and TensorFlow (v1.15.0, Google Inc.). All the image registration algorithms are implemented with MATLAB r2019a. For the training of our models, we used a desktop computer with a dual GTX 1080 Ti graphical processing unit (GPU, Nvidia Inc.) and Intel^®^ Core^TM^ i7-8700 central processing unit (CPU, Intel Inc.) and 64 GB of RAM, running Windows 10 operating system (Microsoft Inc.). The typical training time of the convolutional neural networks used in our registration process and the pseudo-H&E virtual staining network (i.e., networks *A*, *A*′*, B*, and VS_HE_) is ~24 h when using a single GPU. For our acetic acid virtual staining network (i.e., VS_AA_), the typical training time for using a single GPU is ~72 h. Once the VS_AA_ and VS_HE_ networks are trained, using the same computer with two GTX 1080 Ti GPUs we can execute the model inference at a speed of ~0.2632 and ~0.0818 s for an image size of 896 × 896-pixels, respectively. Using a more powerful machine with eight Tesla A100 GPUs, the virtual staining speed can be substantially increased to ~0.0173 and ~0.0046 s per image (896 × 896-pixels), for VS_AA_ and VS_HE_ networks, respectively.

## Supplementary information

Supplementary Information

Supplementary Video 1

Supplementary Video 2

Supplementary Video 3

Supplementary Video 4

## Data Availability

The authors declare that all data supporting the results reported in this study are available within the paper and the Supplementary Information. Additional data used for the study are available from the corresponding author upon reasonable request.

## Code availability

All the deep-learning models used in this work employ standard libraries and scripts that are publicly available in TensorFlow. Supplementary Information accompanies the manuscript on the Light: Science & Applications website (http://www.nature.com/lsa)
