# Supplementary material for: Biopsy-free in vivo virtual histology of skin using deep learning
Source: Light Sci Appl. 2021 Nov 18;10:233. doi: 10.1038/s41377-021-00674-8 (PMC8602311; doi:10.1038/s41377-021-00674-8)
Supplement: Supplementary file 1 — Supplementary Information [file 41377_2021_674_MOESM1_ESM.pdf]

Supplementary Information for

Biopsy-free *in vivo* virtual histology of skin using deep learning

Jingxi Li[#,1,2,3], Jason Garfinkel[#,4], Xiaoran Zhang[1], Di Wu[5], Yijie Zhang[1,2,3], Kevin De Haan[1,2,3], Hongda Wang[1,2,3], Tairan Liu[1,2,3], Bijie Bai[1,2,3], Yair Rivenson[1,2,3], Gennady Rubinstein[*,4], Philip O. Scumpia[*,6,7], Aydogan Ozcan[*,1,2,3,8]

[1]Electrical and Computer Engineering Department, University of California, Los Angeles, CA, 90095, USA.

[2]Bioengineering Department, University of California, Los Angeles, CA, 90095, USA.

[3]California NanoSystems Institute (CNSI), University of California, Los Angeles, CA, 90095, USA.

[4]Dermatology and Laser Centre, Studio City, CA, 91604, USA

[5]Computer Science Department, University of California, Los Angeles, CA, 90095, USA.

[6]Division of Dermatology, University of California, Los Angeles, CA, 90095, USA.

[7]Department of Dermatology, Veterans Affairs Greater Los Angeles Healthcare System, Los Angeles, CA, 90073, USA.

[8]Department of Surgery, University of California, Los Angeles, CA, 90095, USA

[#] These authors contributed equally to the work

*Address Correspondence to:

Aydogan Ozcan, PhD, email; ozcan@ucla.edu

Philip Scumpia, MD PhD, email; pscumpia@mednet.ucla.edu

Gennady Rubinstein, MD, email; grmd@sbcglobal.net

This document contains Supplementary Note 1, Supplementary Figures S1-9, and Supplementary Videos 1-4.

Supplementary Note 1: Pyramid elastic registration algorithm

The specific procedures of the pyramid elastic registration algorithm are detailed in the following pseudo-code:

Algorithm Pyramid elastic registration

Set the block size shrinking speed α, the minimum block size β, the shift error tolerance ε;
Initialize the number of blocks N using N_0;
Input the image pair I_A and I_B to be cross-registered;
Repeat:
  Divide both I_A and I_B into $N \times N$ image blocks;
  Repeat:
    Calculate the 2D cross-correlation map CCM between the corresponding blocks in I_A and I_B;
    Calculate the shift amount of each pair of blocks through fitting a 2D Gaussian function to the peak of the CCM (see details below), forming a 2D shift map SM composed of $N \times N$ elements;
    Perform linear interpolation of SM to fit the image size of I_A (I_B), producing SM';
    Register I_B to I_A based on SM', creating a new I_B;
  Until the maximum value of the SM is smaller than the shift error tolerance ε;
  $N \leftarrow$ round($N \times \alpha$);
Until the block size is smaller than β;
Result: I_B is finely registered to I_A with sub-pixel level accuracy.

In our paper, for performing this elastic registration, the values of α, β, ε and N_0 are empirically set as 1.4, 50, 0.5 and 3, respectively. The detailed procedures of calculating the 2D shift map SM based on the 2D cross-correlation map CCM can be summarized as:

1) Calculate the normalized cross-correlation map $nCCM$, which is defined as

$$nCCM = \frac{CCM - min(CCM)}{max(CCM) - min(CCM)} \times \left(max(PCC) - min(PCC)\right) + min(PCC),$$

where CCM is the cross-correlation map, defined as

$$CCM(u,v) = \sum_{x,y} \left[f(x,y) - \bar{f}\right]\left[g(x-u, y-v) - \bar{g}\right],$$

where f and g represent two images, and $\bar{a}$ refers to the two-dimensional mean operator of an image, a. The locations of the maximum and minimum values of CCM indicate the most likely and the most unlikely (respectively) relative shifts of the images. PCC refers to the Pearson correlation coefficient of the two images, and its definition can be found in Materials and Methods, sub-section "Quantitative morphological analysis of virtual staining results" of the main text.

2) The normalized cross-correlation map $nCCM$ is then fit to a 2D Gaussian function, which is defined as:

$$G(x,y) = A \cdot \exp\left(-\left(\frac{(x-x_0)^2}{2\sigma_x^2} + \frac{(y-y_0)^2}{2\sigma_y^2}\right)\right),$$

where x_0 and y_0 represent the lateral position of the peak that indicates the shift amount between the two images along the x and y directions, respectively, and A represents the similarity of the two images, f and g.

Supplementary Figures

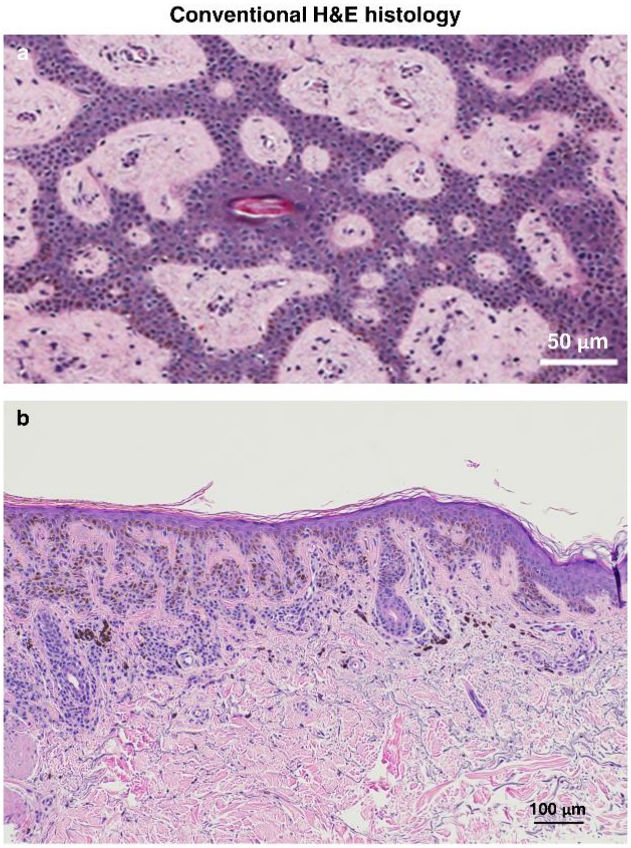

Figure S1. Histology image example for comparison. a, Bright-field H&E image of a skin tissue section cut *horizontally* through the dermal-epidermal junction. **b,** Bright-field H&E image of a skin tissue section cut *vertically*. Pigmented melanocytes that are stained dark brown can be clearly observed.

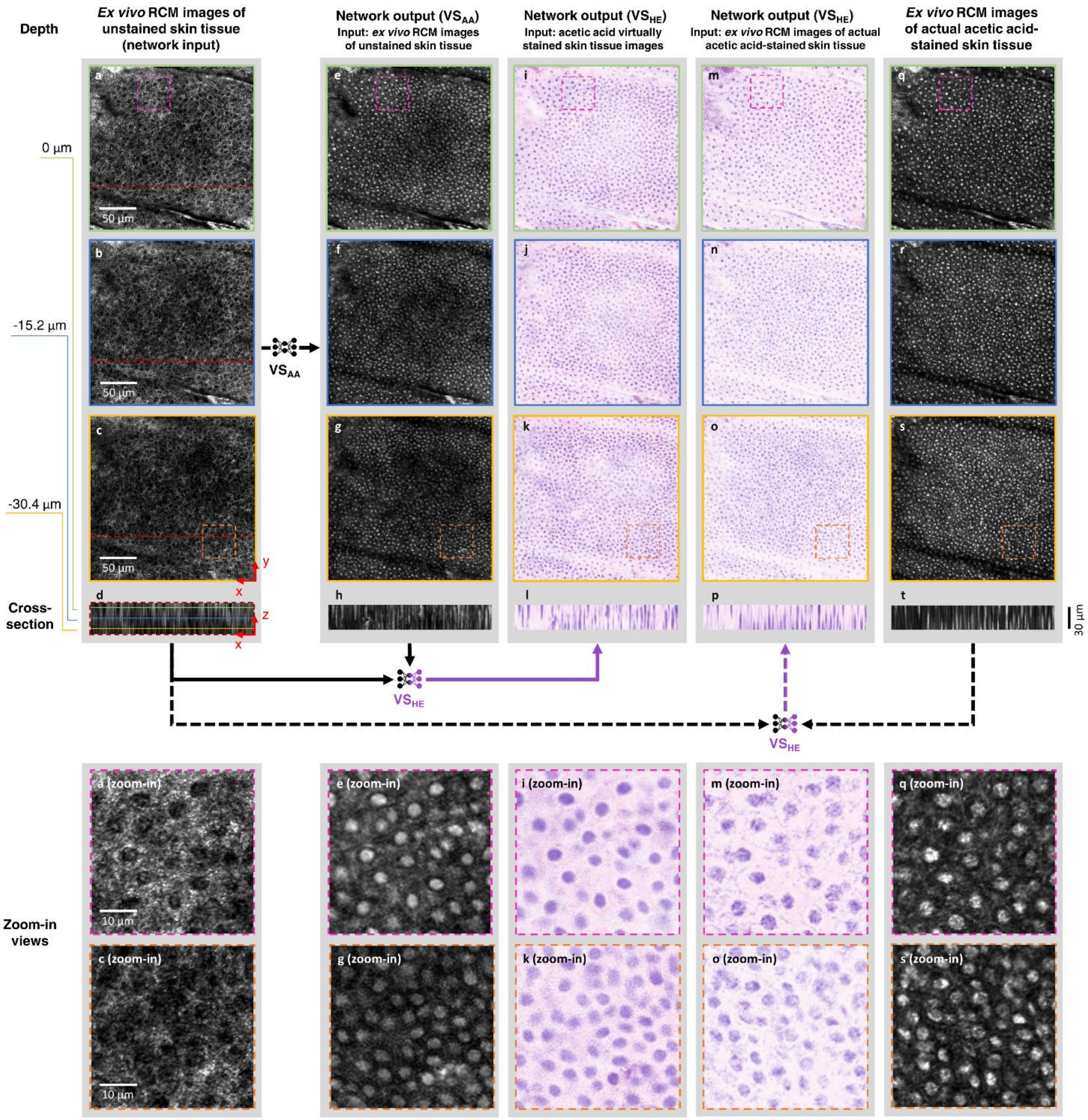

Figure S2. 3D *ex vivo* virtual staining results of normal skin tissue and comparison with ground truth with actual acetic acid staining. a-c, Label-free RCM images showing an *ex vivo* normal skin tissue area at different depths without any staining, which serve as the network inputs. The axial depths of (b) and (c) were 15.2 and 30.4 μm below the depth of (a), into the skin, respectively. **d,** Cross-section of the RCM image stack of the tissue area including (a)-(c). Lines in different colors are used to indicate the depth positions of (a)-(c). **e-g,** Acetic acid virtual staining results of the same tissue areas and depths as in (a)-(c) generated by VS_{AA}. **h** is the image stack cross-section of the acetic acid virtual staining results including (e)-(g). **i-k,** Pseudo-H&E virtual staining results generated using the acetic acid virtually stained tissue images (e)-(g). These H&E images were generated by VS_{HE} that took both the RCM

images of the unstained tissue (a)-(c) and acetic acid virtually stained tissue images (e)-(g) as input (see solid arrows at the bottom). **l,** Cross-section of the pseudo-H&E virtually stained tissue image stack including (i)-(k) generated using the acetic acid virtually stained tissue images. **q-s,** RCM images of the same tissue area and depth as in (a)-(c) after the actual acetic acid staining process, which serve as ground truth for (e)-(g). **t** shows the cross-section of the image stack of the tissue stained with acetic acid including (q)-(s). **m-o,** Pseudo-H&E virtual staining results generated using the actual acetic acid-stained tissue images (q)-(s). These H&E images were generated by the same VS_{HE} that took the RCM images of the unstained tissue (a)-(c) and actual acetic acid-stained images (q)-(s) as input (see dashed arrows at the bottom, and see the Materials and Methods section, main text, for details). **p** shows the cross-section of pseudo-H&E virtually stained tissue image stack including (m)-(o) generated using the actual acetic tissue acid-stained images. Zoomed-in views of some portions of the images are provided at the bottom for a better visual comparison of details

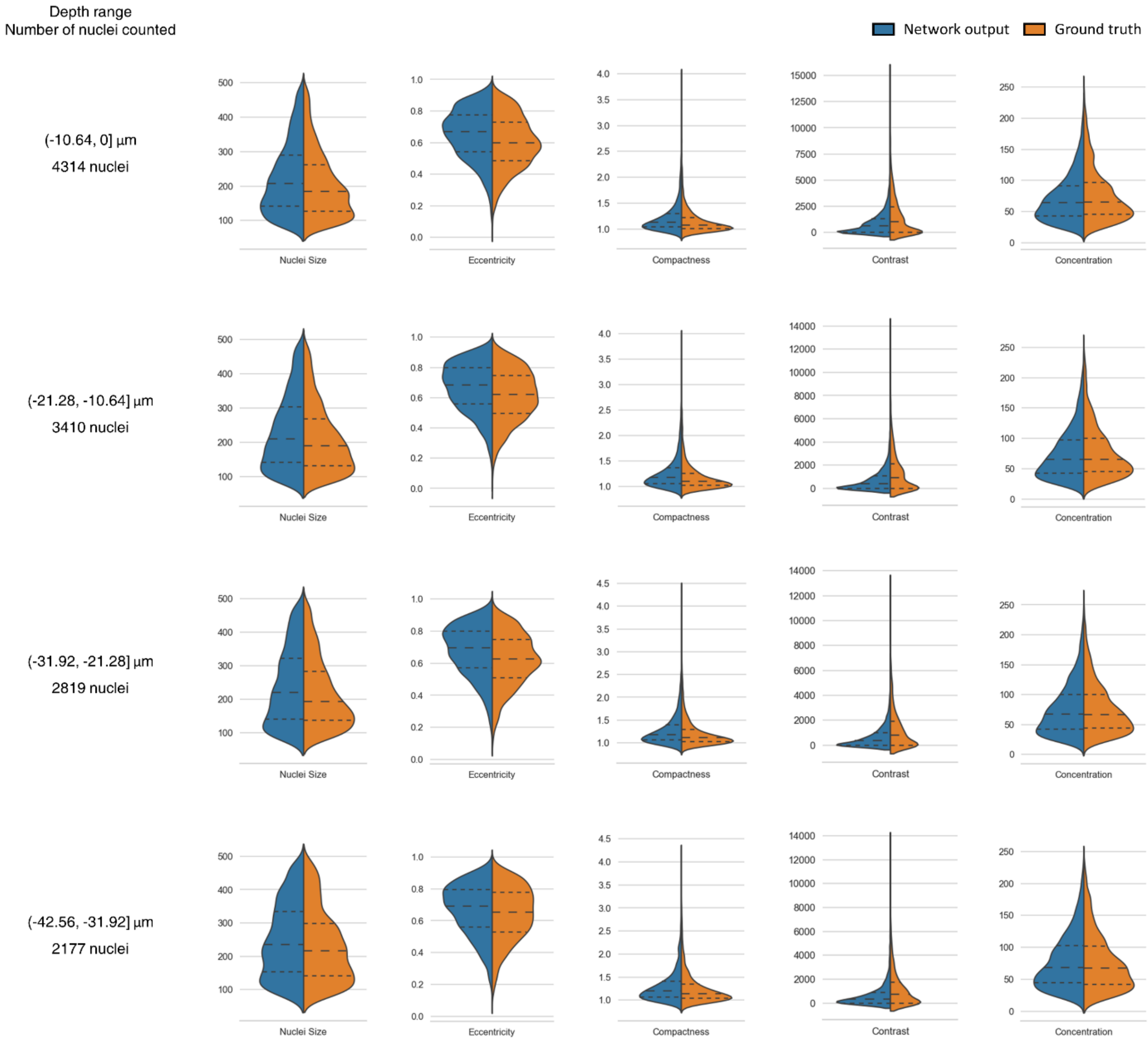

Figure S3. Quantitative morphological analysis of the 3D virtual staining results using the *ex vivo* skin tissue area shown in Figure 4 (main text). The violin plots show comparisons of the statistical distributions of the nuclear morphological profiles between the acetic acid virtually stained skin tissue images (blue) and their corresponding ground truth obtained using the actual acetic acid staining (orange). The image stack is divided into four subsets according to the depth range, each corresponding to one row and covering an axial depth range of 10.64 µm, where statistical analyses were performed individually using five metrics including: nuclei size, eccentricity, compactness, contrast, and concentration. Dashed lines in the violin plots from top to bottom represent the 75%, 50% (median) and 25% quartile, respectively. The nuclear object detection was performed using the segmentation algorithm in CellProfiler (see the Materials and Methods section, main text, for details).

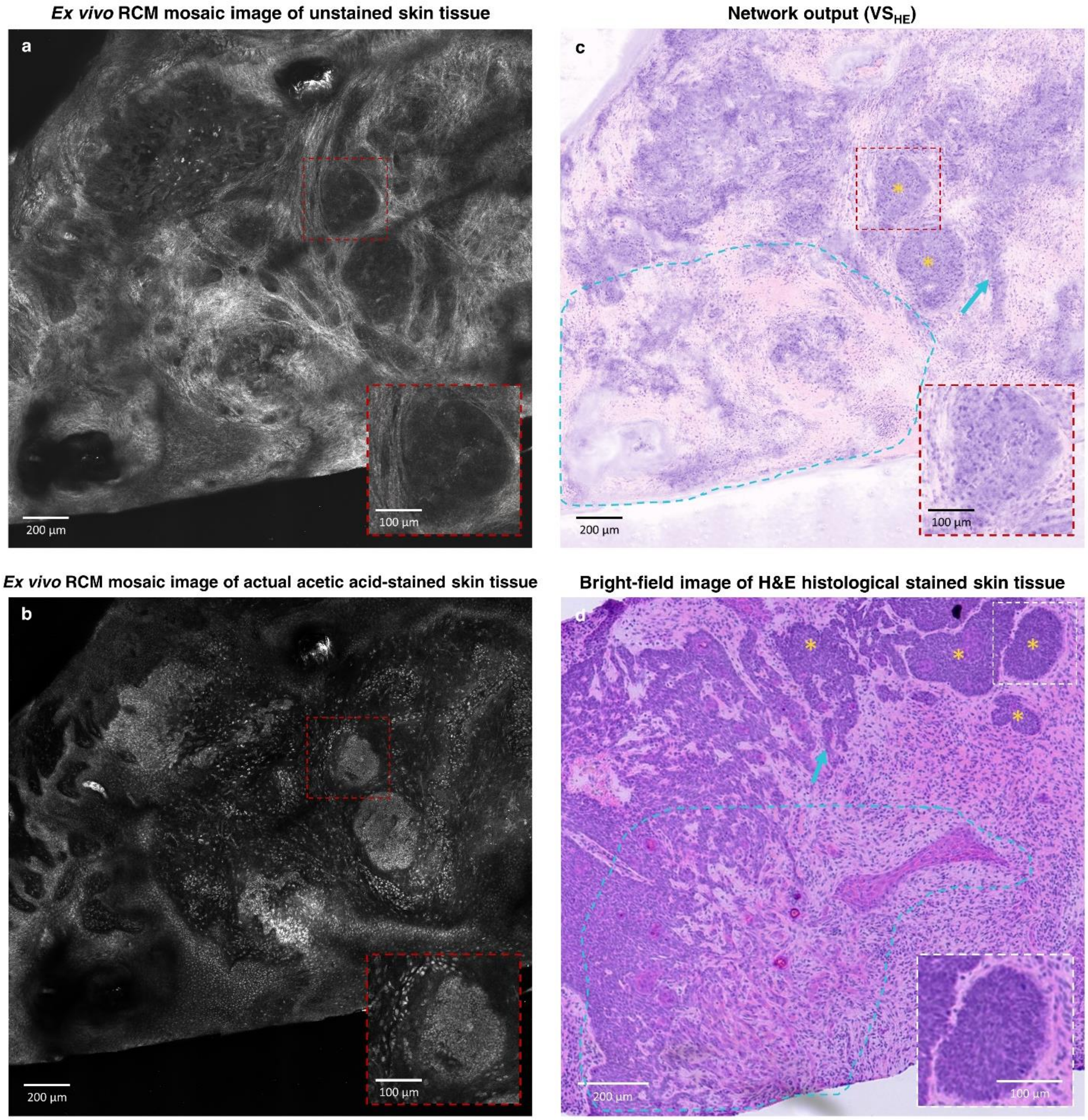

Figure S4. Pseudo-H&E virtual staining result of a mosaic image of an *ex vivo* skin lesion containing BCC, compared with mosaic RCM image of the same tissue stained with acetic acid and bright-field H&E image of the same tissue with frozen section histology. a, Large field-of-view RCM image mosaic showing an *ex vivo* skin lesion containing BCC. **b,** RCM image mosaic of the same tissue lesion in (a) but stained with acetic acid. **c,** Pseudo-H&E virtual staining result of (a). **d,** Bright-field image of the same tissue lesion in (a) after the histological H&E staining process. Note that tissue was processed per Mohs micrographic surgery for margin control, which results in the tissue being in a different plane than horizontal/en face in order to visualize the deep and lateral tissue margins simultaneously. Yellow asterisks highlight tumor nodules. Individual cords of BCC are highlighted by blue islands

(marked with light blue arrows). Anastomosing cords of BCC with focal keratinization (bright pink appearance of keratin within basaloid cells on H&E) and similar corresponding areas on virtual histology are shown within the light blue dotted region.

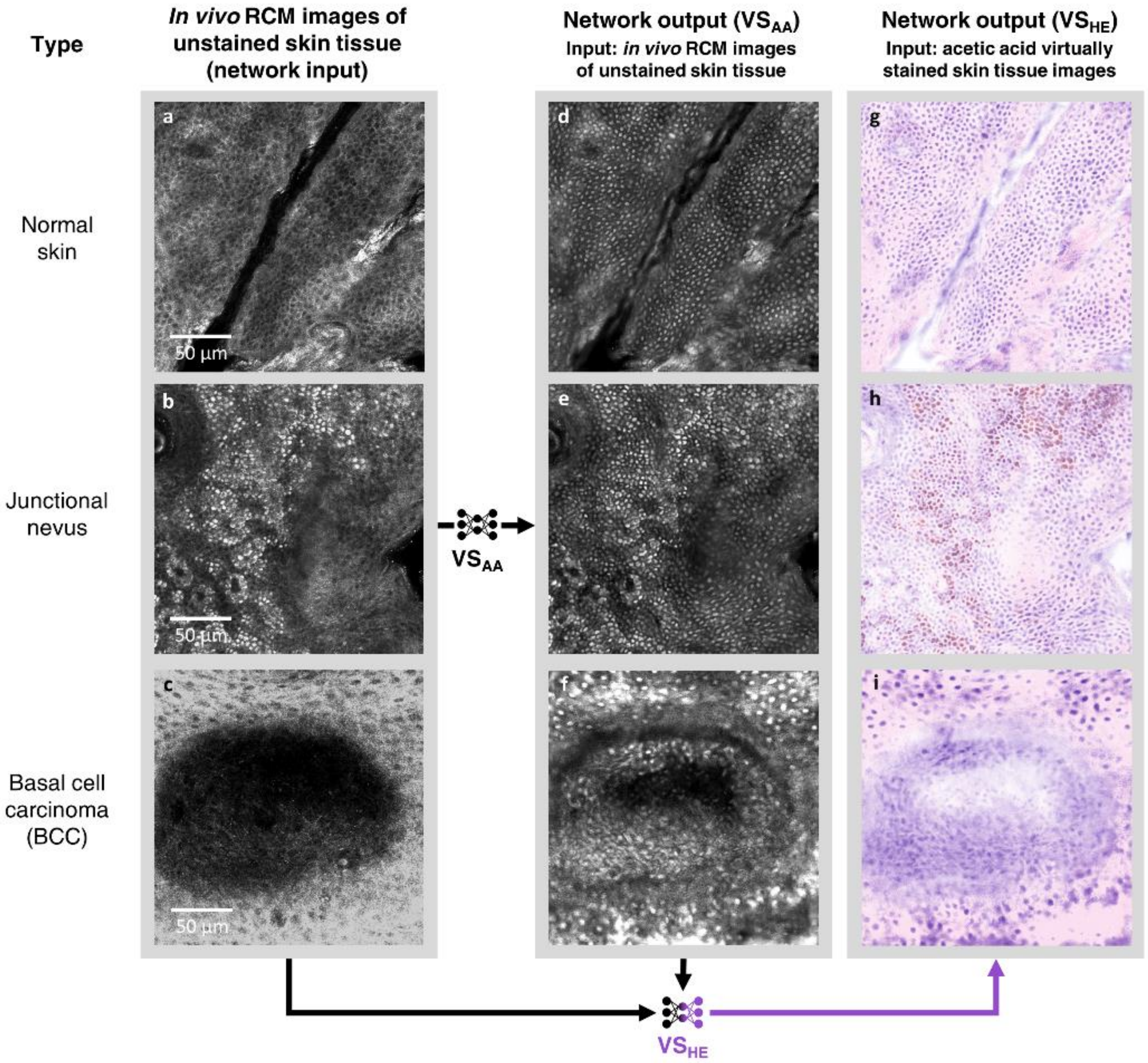

Figure S5. Virtual staining results for different types of *in vivo* skin tissue areas. a-c, Label-free RCM images of three different types of *in vivo* skin tissue areas, including (a) normal skin, (b) junctional nevus containing melanocytes and (c) skin containing basal cell carcinoma (BCC), which are used as the network inputs. **d-f,** Acetic acid virtual staining results of the same tissue areas in (a)-(c) generated by the deep neural network VS_{AA}. **g-i,** Pseudo-H&E virtual staining results generated using the acetic acid virtually stained tissue images (d)-(f). These H&E images were generated by the virtual staining network VS_{HE} that took both the RCM images of the unstained tissue (a)-(c) and the acetic acid virtually stained tissue images (d)-(f) as input (see the arrows at the bottom, and the Materials and Methods section, main text, for details).

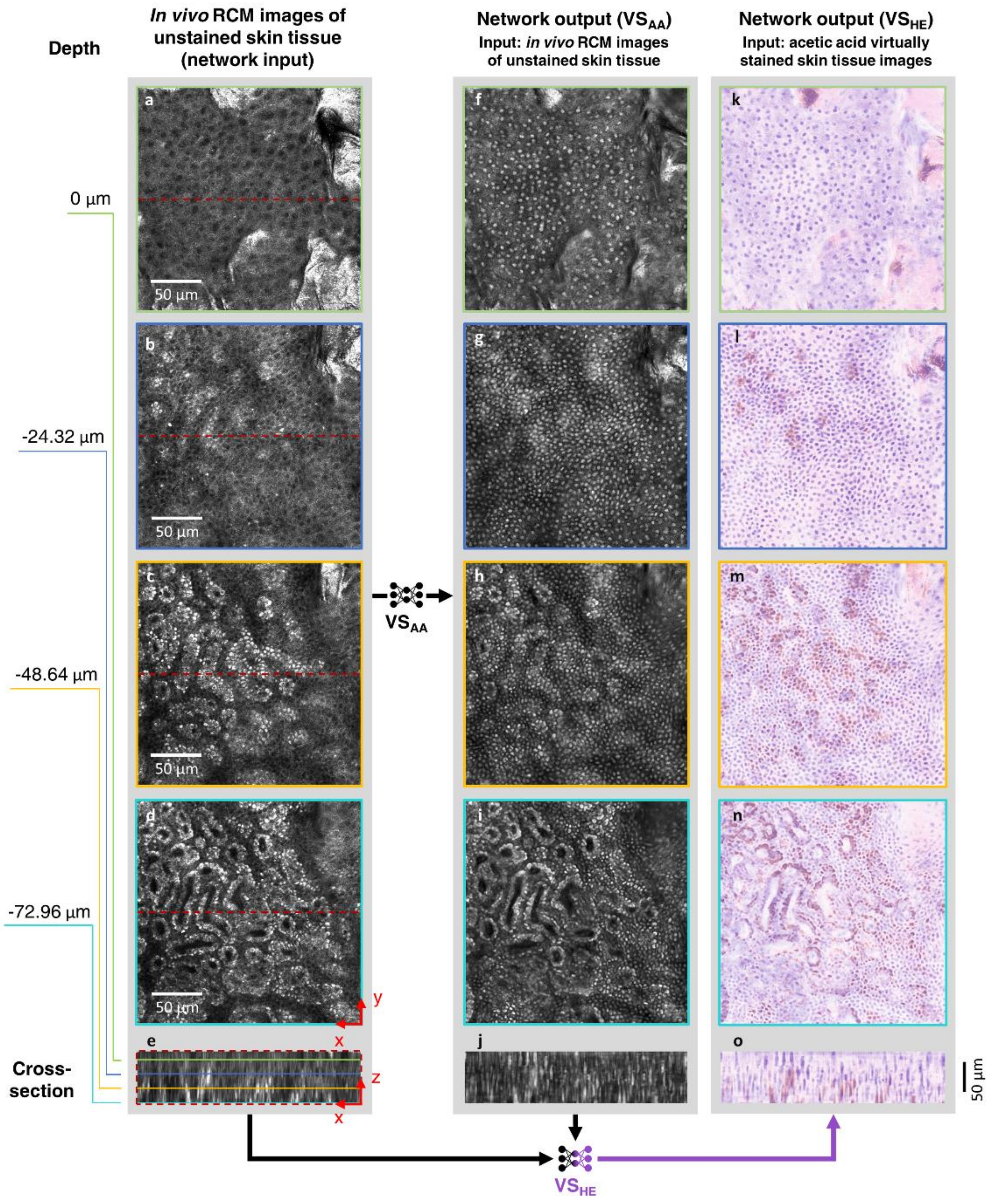

Figure S6. 3D *in vivo* virtual staining results of skin tissue. a-d, Label-free RCM images showing an *in vivo* skin tissue area at different depths without any staining, serving as the network inputs. The depths of (b), (c) and (d) were 24.32, 48.64 and 72.96 µm below the depth of (a), into the skin, respectively. **e,** Cross-section of the RCM image stack

of the tissue area including (a)-(d). Lines in different colors are used to indicate the depth positions of (a)-(d). **f-i,** Acetic acid virtual staining results of the same tissue area and depth as in (a)-(d) generated by VS_{AA}. **j** is the image stack cross-section of the acetic acid virtual staining results including (f)-(i). **k-n,** Pseudo-H&E virtual staining generated using the acetic acid virtually stained tissue images (f)-(i). These H&E images were generated by the virtual staining network VS_{HE} that took both the RCM images of the unstained tissue (a)-(d) and the acetic acid virtually stained tissue images (f)-(i) as input (see arrows at the bottom, and the Materials and Methods section, main text, for details). **o,** Cross-section of the pseudo-H&E virtually stained tissue image stack including (k)-(n) generated using the acetic acid virtually stained tissue images.

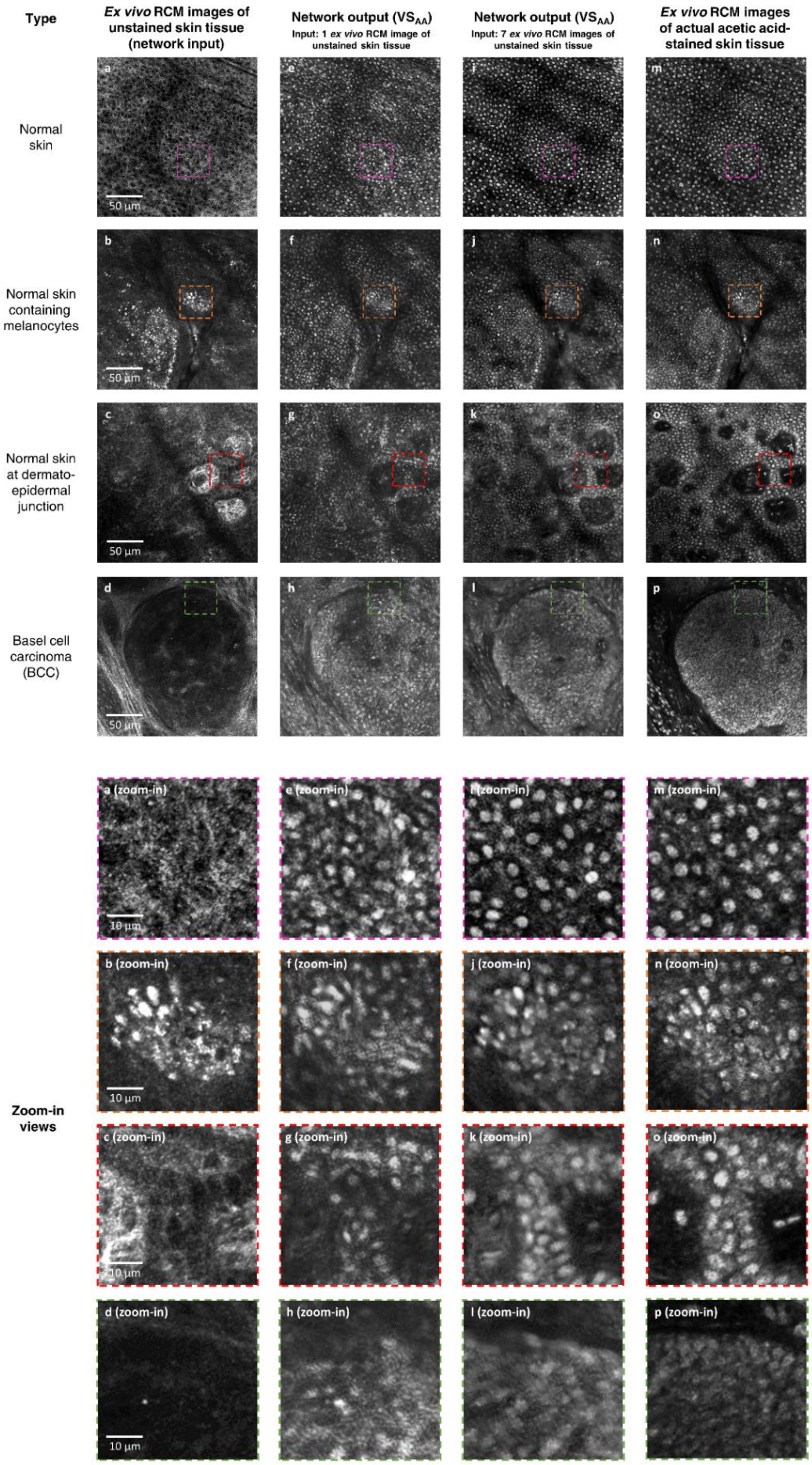

Figure S7. Comparison of the results obtained by the acetic acid virtual staining networks using 2D and 3D

input. a-d, Label-free RCM images of four different types of *ex vivo* skin tissue areas, including (a) normal skin, (b) normal skin containing melanocytes, (c) normal skin at the dermal-epidermal junction and (d) skin containing BCC, which are used as network inputs. **e-h,** Acetic acid virtual staining results of the same tissue areas in (a)-(c), which are generated by a trained virtual staining network taking only *one* label-free RCM image as input. **i-l,** Same as (e)-(h) but the images are generated by a trained virtual staining network taking 7 label-free RCM images that are axially neighbored as input. **m-p,** *Ex vivo* RCM images of the same tissue area and depths as in (a)-(d) after the actual acetic acid staining process, serving as the ground truth for (e)-(h) and (i)-(l). Zoomed-in views of some portions of the images are provided at the bottom for a better visual comparison of details

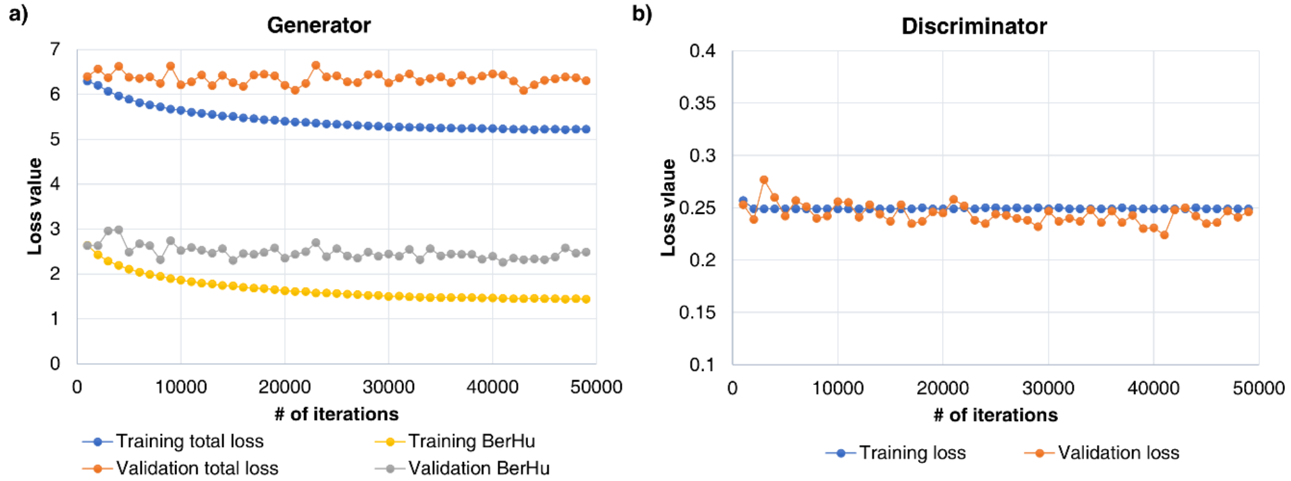

Figure S8. Loss curves during the training of the virtual staining neural network. a, Generator loss as a function of the number of iterations. **b,** Discriminator loss as a function of the number of iterations.

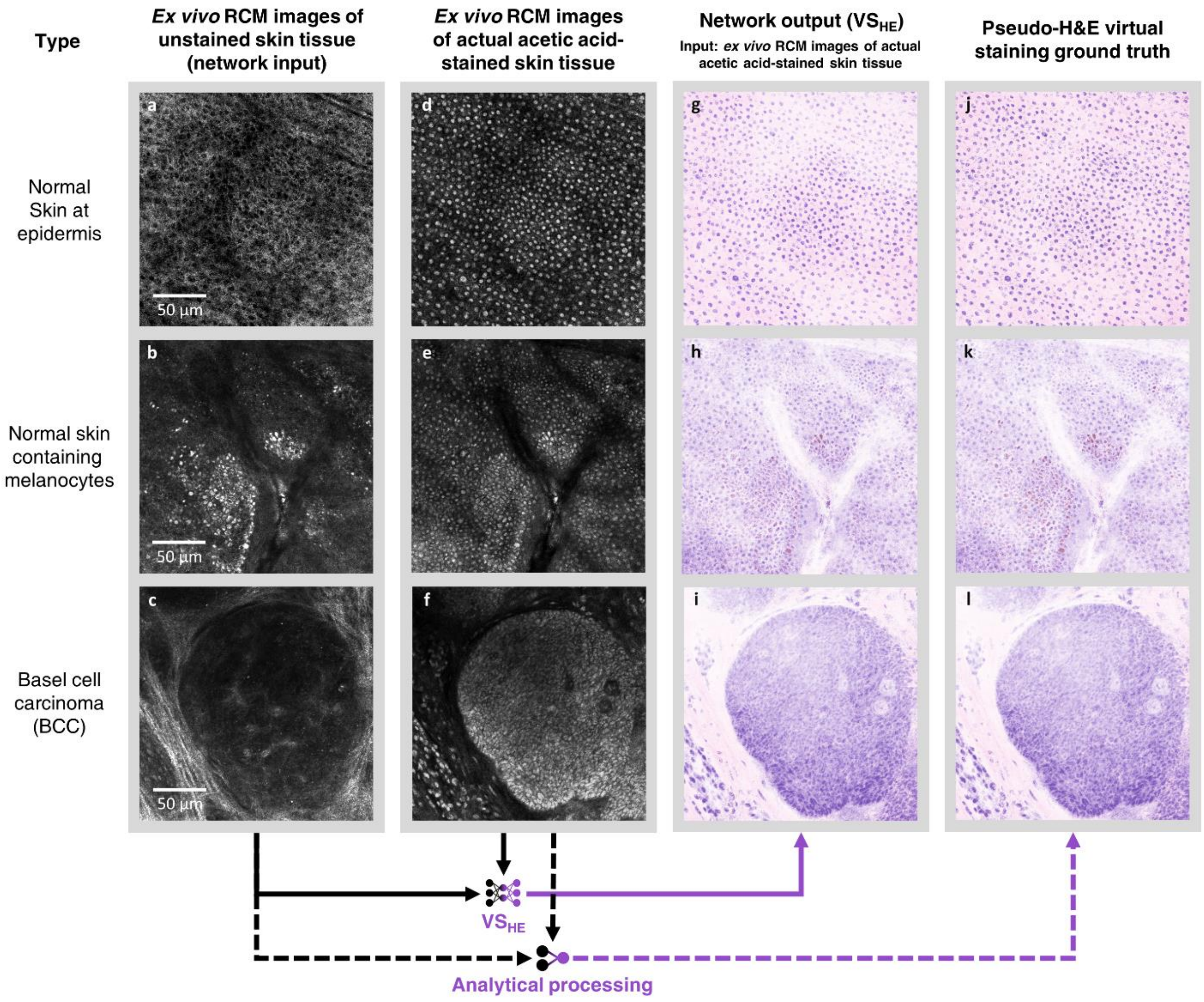

Figure S9. Comparison of the pseudo-H&E virtual staining results against their ground truth generated by analytical processing. a-c, Label-free RCM images of three different types of *ex vivo* skin tissue areas, including (a) normal skin, (b) a melanocytic nevus and (c) skin containing BCC, which are used as input of the virtual staining neural networks. **d-f,** RCM images of the same tissue area and depth as (a)-(c) after the actual acetic acid staining process. **g-i,** Pseudo-H&E virtual staining results generated using the actual acetic acid-stained images (d)-(f). These H&E images were generated by the same virtual staining network VS_{HE} that took the RCM images of the unstained tissue (a)-(c) and actual acetic acid-stained images (d)-(f) as input (see solid arrows). **j-l,** Pseudo-H&E ground truth images for (g)-(i), which were generated by analytical processing (using Eq. (8) in the Materials and Methods section).

Supplementary Videos

Supplementary Videos 1&2. (Left) Examples of label-free RCM stack images of skin tissue acquired immediately from our RCM device with relatively *mild* sample motion observed during the imaging process. **(Right)** Successfully registered versions of the same images shown on the left using the elastic stack registration algorithm described in the Materials and Methods section and Supplementary Note 1.

Supplementary Videos 3&4. (Left) Examples of label-free RCM stack images of skin tissue acquired immediately from our RCM device with relatively *strong* sample motion observed during the imaging process. **(Right)** Successfully registered versions of the same images shown on the left using the elastic stack registration algorithm described in the Materials and Methods section and Supplementary Note 1. The last frame of the video indicates the depth level after which the registration algorithm started to fail due to strong sample motion during image acquisition.
